# Supplementary material for: Combined Vorinostat and Chloroquine Inhibit Sodium Iodide Symporter Endocytosis and Enhance Radionuclide Uptake In Vivo
Source: Clin Cancer Res. Author manuscript; Available in PMC 2024 Apr 1. (PMC7615786; doi:10.1158/1078-0432.CCR-23-2043)
Supplement: Supplementary Figure S1 [file EMS190879-supplement-Supplementary_Figure_S1.pdf]

SUPPLEMENTARY FIGURE S1

A TPC-1

Laboratory Report

Test Requested	Cell Line Authentication
Case Number	C-25115d
Date Sample Received	08/04/2022
Date Sample Tested	11/04/2022
Date Sample Reported	19/04/2022

Sample Name	Sample/Comparison Profile Source	Sample Number	DNA Number
TPC-1	University of Birmingham	S-1057716	D-1057716
TPC-1	Cellosaurus Database	N/A	N/A

Table of Allelic Data

STR Locus	Genotypes: TPC-1 (Test Sample)	Genotypes: TPC-1 (Comparison Sample)	Match vs. Mis-Match
D5	8 | 10	8 | 10	Match
D13	11 | 12	11 | 12	Match
D7	11 | 11	11 | 11	Match
D16	9 | 9	9 | 9	Match
vWA	14 | 18	14 | 18	Match
Amel	X | X	X | X	Match
TPOX	11 | 11	11 | 11	Match
CSF1PO	11 | 12	11 | 12	Match
THO1	9 | 9	9 | 9	Match

Matching Percentage: **100%**
Outcome: **Related**

B 8505C

Laboratory Report

Test Requested	Cell Line Authentication
Case Number	C-25115e
Date Sample Received	08/04/2022
Date Sample Tested	11/04/2022
Date Sample Reported	19/04/2022

Sample Name	Sample/Comparison Profile Source	Sample Number	DNA Number
8505C	University of Birmingham	S-1057717	D-1057717
8505C	DSMZ Database	N/A	N/A

Table of Allelic Data

STR Locus	Genotypes: 8505C (Test Sample)	Genotypes: 8505C (Comparison Sample)	Match vs. Mis-Match
D5	10 | 11	10 | 11	Match
D13	13 | 13	13 | 13	Match
D7	10 | 10	10 | 10	Match
D16	12 | 12	12 | 12	Match
vWA	17 | 19	17 | 19	Match
Amel	X | X	X | X	Match
TPOX	10 | 11	10 | 11	Match
CSF1PO	12 | 13	12 | 13	Match
THO1	6 | 9	6 | 9	Match

Matching Percentage: **100%**
Outcome: **Related**

C HeLa

Laboratory Report

Test Requested	Cell Line Authentication
Case Number	C-25115f
Date Sample Received	08/04/2022
Date Sample Tested	11/04/2022
Date Sample Reported	19/04/2022

Sample Name	Sample/Comparison Profile Source	Sample Number	DNA Number
HeLa	University of Birmingham	S-1057718	D-1057718
HeLa	DSMZ Database	N/A	N/A

Table of Allelic Data

STR Locus	Genotypes: HeLa (Test Sample)	Genotypes: HeLa (Comparison Sample)	Match vs. Mis-Match
D5	11 | 12	11 | 12	Match
D13	12 | 13.3	12 | 13.3	Match
D7	8 | 12	8 | 12	Match
D16	9 | 10	9 | 10	Match
vWA	16 | 18	16 | 18	Match
Amel	X | X	X | X	Match
TPOX	8 | 12	8 | 12	Match
CSF1PO	9 | 10	9 | 10	Match
THO1	7 | 7	7 | 7	Match

Matching Percentage: **100%**
Outcome: **Related**

D HEK293

Laboratory Report

Test Requested	Cell Line Authentication
Case Number	C-25115h
Date Sample Received	08/04/2022
Date Sample Tested	11/04/2022
Date Sample Reported	19/04/2022

Sample Name	Sample/Comparison Profile Source	Sample Number	DNA Number
HEK293	University of Birmingham	S-1057720	D-1057720
HEK-293.2sus	DSMZ Database	N/A	N/A

Table of Allelic Data

STR Locus	Genotypes: HEK293 (Test Sample)	Genotypes: HEK-293.2sus (Comparison Sample)	Match vs. Mis-Match
D5	8 | 8	8 | 8	Match
D13	12 | 14	12 | 14	Match
D7	11 | 11	11 | 12	Mis-Match
D16	9 | 13	9 | 13	Match
vWA	16 | 19	16 | 19	Match
Amel	X | X	X | X	Match
TPOX	11 | 11	11 | 11	Match
CSF1PO	12 | 12	12 | 12	Match
THO1	7 | 9.3	7 | 9.3	Match

Matching Percentage: **94%**
Outcome: **Related**

Figure S1. Representative STR profiles of cell lines. Examples of cell line authentication reports for **A,** TPC-1, **B,** 8505C, **C,** HeLa and **D,** HEK293 cells generated by NorthGene (Biofortuna). DNA profiles were compared to profiles located on either the Cellosaurus or DSMZ database as indicated. Analysis was conducted using 8 loci allowing identical matches and approximate power of discrimination of 1 in 1,000,000,000. Cell lines were considered to be related with a matching percentage > 80%.
