# Supplementary material for: Combined Vorinostat and Chloroquine Inhibit Sodium Iodide Symporter Endocytosis and Enhance Radionuclide Uptake In Vivo
Source: Clin Cancer Res. Author manuscript; Available in PMC 2024 Apr 1. (PMC7615786; doi:10.1158/1078-0432.CCR-23-2043)
Supplement: Supplementary Figure S2 [file EMS190879-supplement-Supplementary_Figure_S2.pdf]

SUPPLEMENTARY FIGURE S2

A

Gene	BRAF-like, RAI (n = 137, FDR < 12%)		BRAF-like, Non-RAI (n = 94)	
	p	N_{REC}	p	N_{REC}
ACTB	0.593	12,9	0.985	1,1
ACTR3	0.804	11,10	0.146	2,0
AP2A2	0.043	15,6	0.956	1,1
AP2B1	0.295	13,8	0.969	1,1
ARF6	0.031	15,6	0.146	2,0
ARPC4	0.803	11,10	0.969	1,1
CTTN	0.032	15,6	0.146	2,0
EHD1	0.929	11,10	0.981	1,1
HLA-A	0.009	16,5	0.998	1,1
PDCD6IP	0.871	10,11	0.905	2,0
PICALM	0.432	9,12	0.134	2,0
RAB5C	0.002	17,4	0.956	1,1
SRC	0.126	14,7	0.905	1,1
TF	0.228	8,13	0.969	1,1

B

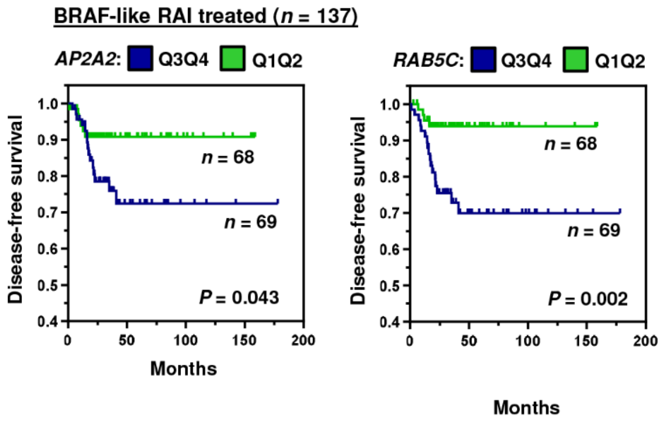

C

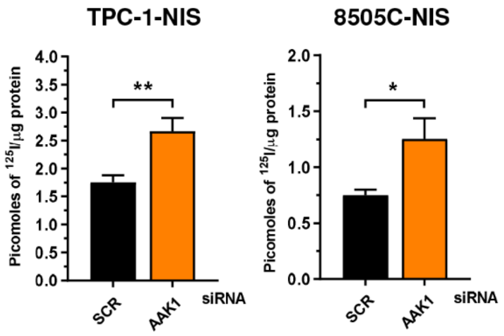

D

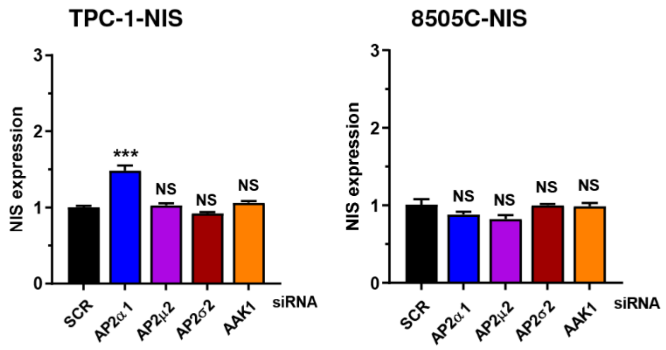

E

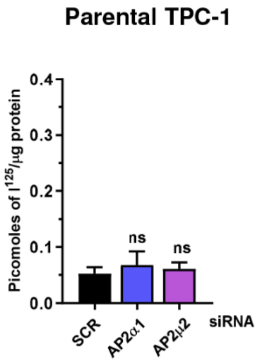

F

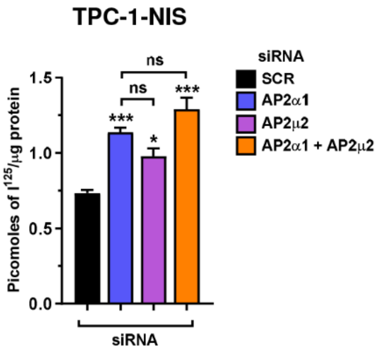

G 8505C-NIS

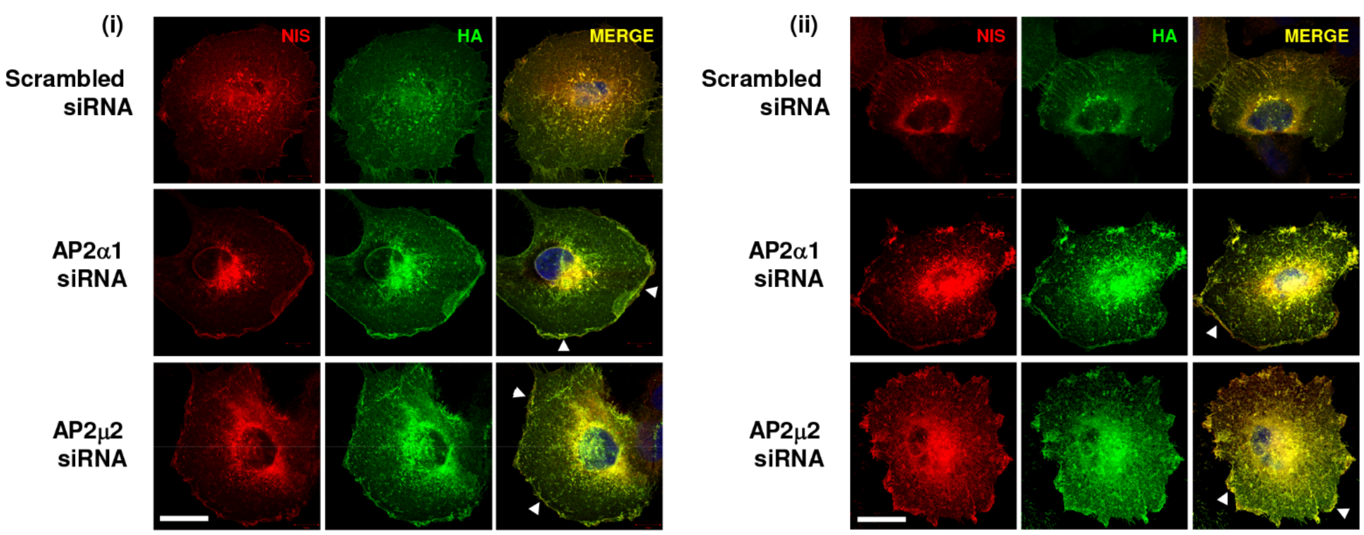

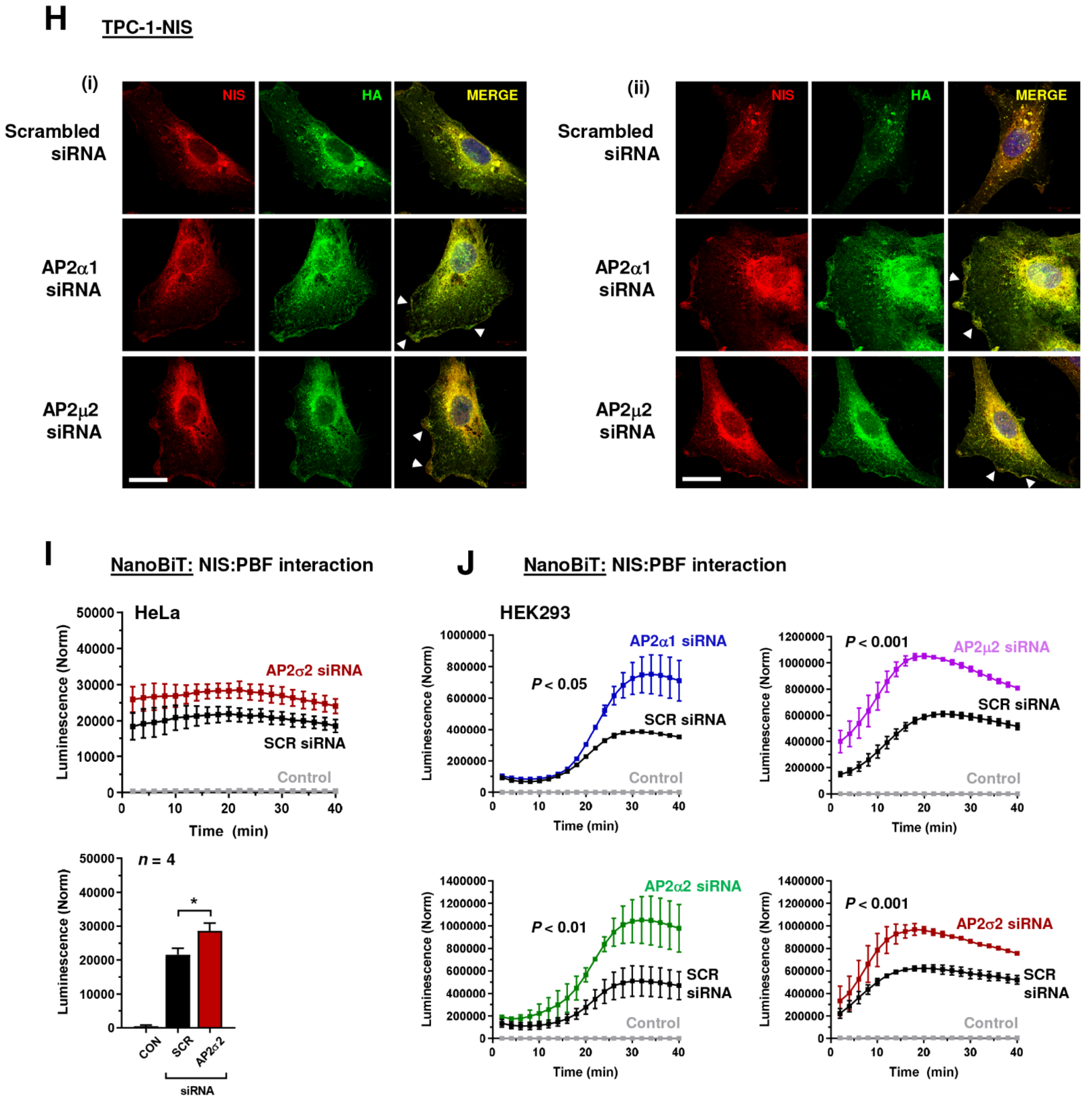

Figure S2. Association of NIS interactors with recurrence. **A,** Kaplan-Meier analysis of RAI-treated (n = 137) and non-RAI treated (n = 94) THCA cohorts stratified on high (Q3Q4) vs low (Q1Q2) tumoral expression; log-rank test. P-values shown with FDR < 12%. N_{REC} = recurrent cases in cohort with high (left) or low (right) tumoral expression. Green = P < 0.05; Yellow = highest percentage of recurrent cases. **B,** Representative Kaplan-Meier analysis of BRAF-like, RAI-treated THCA cohort stratified on high (Q3Q4) vs low (Q1Q2) tumoral expression of *AP2A2* and *RAB5C*; log-rank test. Number (n) of patients per sub-group (high/low) and P-values are shown. **C,** RAI uptake of TPC-1-NIS and 8505C-NIS cells transfected with AAK1 versus scrambled (scr) siRNA. **D,** Relative NIS mRNA levels in TPC-1-NIS and 8505C-NIS cells transfected with siRNA specific for indicated AP2 genes and AAK1. **E** and **F,** RAI uptake of parental TPC-1 (**E**) and TPC-1-NIS (**F**) cells transfected with siRNA specific for indicated AP2 genes. Data presented as mean ± S.E.M., one-way ANOVA followed by Dunnett's (**E**) or Dunnett's Tukey's (**F**) post hoc test (ns, not significant; *P < 0.05; ***P < 0.001). (**G**) Immunofluorescence microscopy of 8505C-NIS cells (i-ii, HA-tagged NIS) transfected with AP2α1, AP2μ2 or scrambled siRNA as indicated. Confocal images represent NIS expression (red), HA expression (green) and a merged image (yellow). Arrows (white) highlight plasma membrane regions with greater NIS localisation. Scale bar – 20 mm. **H**, Same as (**G**) but in TPC-1-NIS cells. **I,** NanoBiT evaluation of protein: protein interaction between NIS and PBF in living HeLa cells transfected with AP2σ2 siRNA. (*bottom*) Normalised NanoBiT assay results at 20 minutes

post-addition of Nano-Glo live cell assay substrate (*n* = 4). **J,** Same as **I** but HEK293 cells transfected with AP2α1, AP2α2, AP2μ2 and AP2σ2 siRNA (*n* = 3 - 4).
