# Supplementary material for: Combined Vorinostat and Chloroquine Inhibit Sodium Iodide Symporter Endocytosis and Enhance Radionuclide Uptake In Vivo
Source: Clin Cancer Res. Author manuscript; Available in PMC 2024 Apr 1. (PMC7615786; doi:10.1158/1078-0432.CCR-23-2043)
Supplement: Supplementary Figure S3 [file EMS190879-supplement-Supplementary_Figure_S3.pdf]

SUPPLEMENTARY FIGURE S3

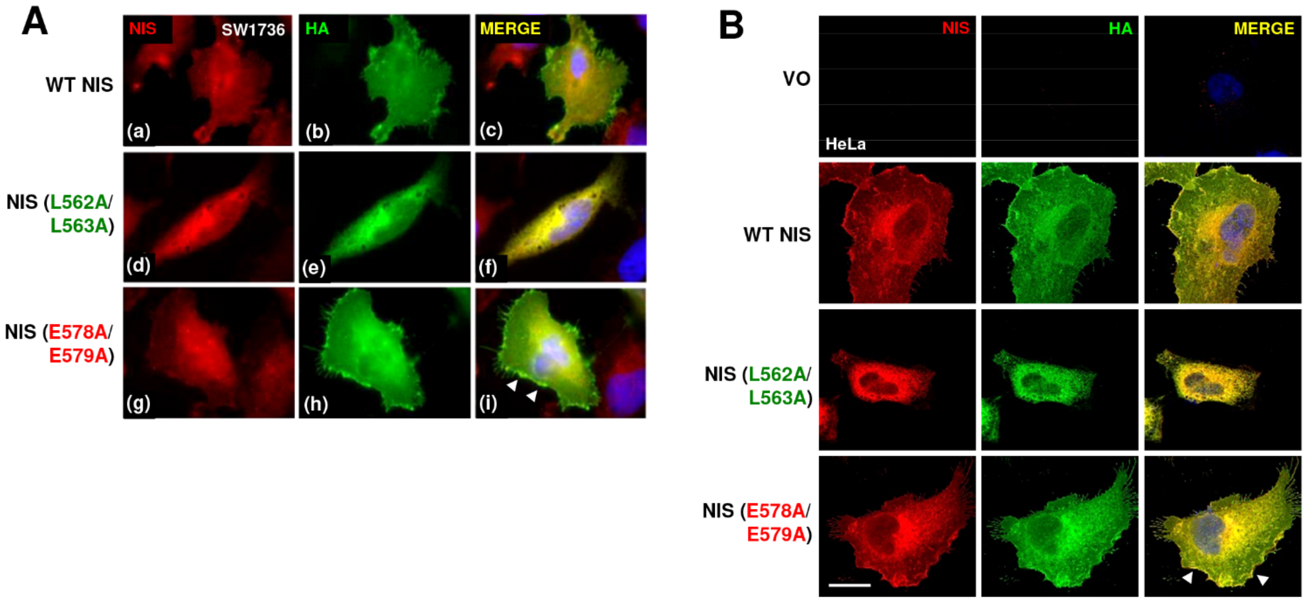

C

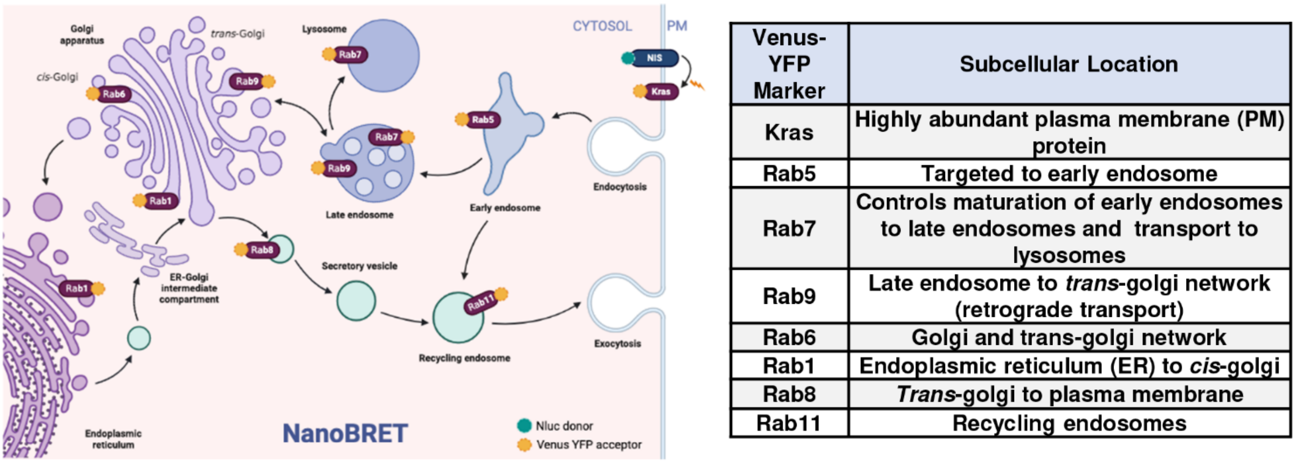

Venus-YFP Marker	Subcellular Location
Kras	Highly abundant plasma membrane (PM) protein
Rab5	Targeted to early endosome
Rab7	Controls maturation of early endosomes to late endosomes and transport to lysosomes
Rab9	Late endosome to *trans*-golgi network (retrograde transport)
Rab6	Golgi and trans-golgi network
Rab1	Endoplasmic reticulum (ER) to *cis*-golgi
Rab8	*Trans*-golgi to plasma membrane
Rab11	Recycling endosomes

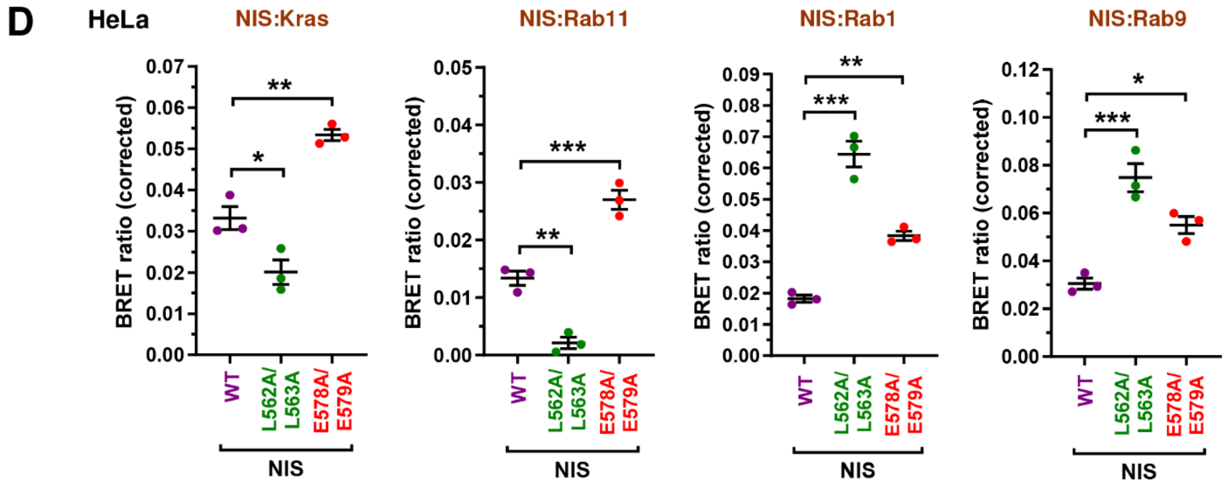

Figure S3. Depletion of APα and μ2 subunits increases NIS at the plasma membrane in thyroid cells. A and **B,** Confocal imaging in SW1736 (**A**) and HeLa (B) cells transfected with HA-tagged wild-type (WT) NIS, NIS mutant L562/L563A or NIS mutant E578A/E579A. Confocal images represent NIS expression (red), HA expression (green) and a merged image (yellow). Arrows (white) highlight PM regions with greater NIS localisation. Scale bar – 20 μm. **C,** Schematic depicting NanoBRET assay to monitor close proximity of NIS with different subcellular markers (e.g. Rab 1, 8 and 11) tagged with Venus. (*right*) Subcellular location of Venus-YFP markers used in study. Created with BioRender.com. **D,** Profiling subcellular location of WT NIS, NIS mutant L562A/L563A and NIS mutant E578A/E579A using the NanoBRET assay. HeLa cells were transiently transfected with NIS or indicated NIS mutant tagged with NLuc,

and the subcellular markers Kras (plasma membrane), Rab11 (recycling endosomes), Rab1 (ER trafficking to cis-golgi) or Rab9 (late endosome to *trans*-golgi network) tagged with Venus.
