# Supplementary material for: Combined Vorinostat and Chloroquine Inhibit Sodium Iodide Symporter Endocytosis and Enhance Radionuclide Uptake In Vivo
Source: Clin Cancer Res. Author manuscript; Available in PMC 2024 Apr 1. (PMC7615786; doi:10.1158/1078-0432.CCR-23-2043)
Supplement: Supplementary Figure S4 [file EMS190879-supplement-Supplementary_Figure_S4.pdf]

SUPPLEMENTARY FIGURE S4

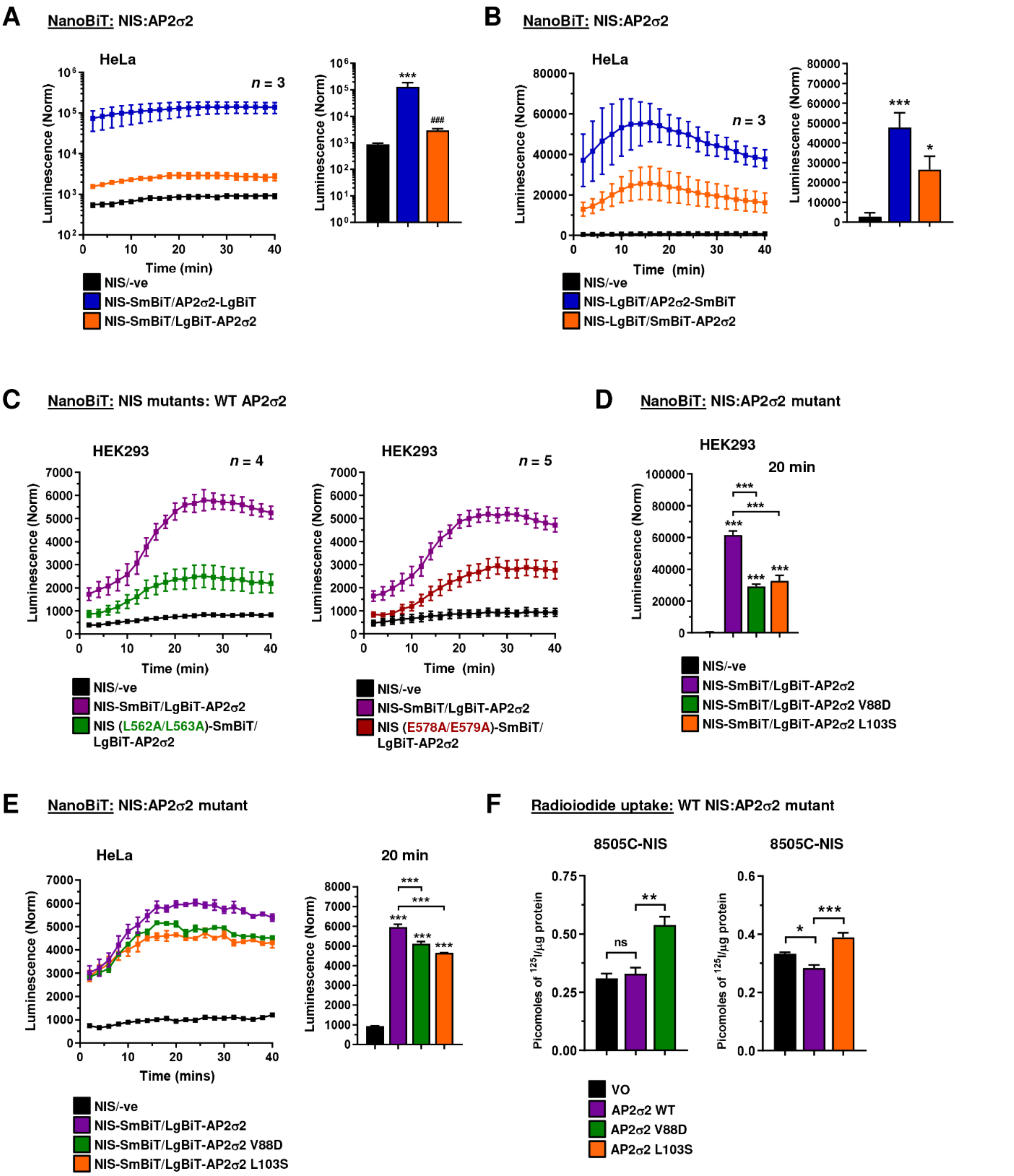

Figure S4. Kinetic profiling of the interaction between NIS and AP2σ2 in live cells. **A,** Live cell kinetic measurement using the NanoBiT assay to evaluate protein-protein interactions between NIS and AP2σ2 tagged with LgBiT in HeLa cells. (*right*) NanoBiT assay results at 20 minutes post-addition of Nano-Glo substrate. **B,** Same as **A** but AP2σ2 tagged with SmBiT. **C,** Same as **A** but using NIS mutants L562A/L563A (*left*) and E578A/E579A (*right*) in HEK293 cells. **D,** Same as **A** but using AP2σ2 mutants V88D and L103S in HEK293 cells. NanoBiT assay results are shown at 20 minutes post-addition of Nano-Glo substrate. **E,** Same as **D** but in HeLa cells. **F,** RAI uptake in 8505C-NIS cells transfected with WT AP2σ2, AP2σ2 mutant V88D and AP2σ2 mutant L103S. Data presented as mean ± S.E.M., n = 4 - 5, one-way ANOVA followed by Tukey's post hoc test (ns, not significant; $^{*}P < 0.05$; $^{**}P < 0.01$; $^{***}P < 0.001$) or unpaired two-tailed t-test ($^{###}P < 0.001$).
