# Supplementary material for: Combined Vorinostat and Chloroquine Inhibit Sodium Iodide Symporter Endocytosis and Enhance Radionuclide Uptake In Vivo
Source: Clin Cancer Res. Author manuscript; Available in PMC 2024 Apr 1. (PMC7615786; doi:10.1158/1078-0432.CCR-23-2043)
Supplement: Supplementary Figure S5 [file EMS190879-supplement-Supplementary_Figure_S5.pdf]

SUPPLEMENTARY FIGURE S5

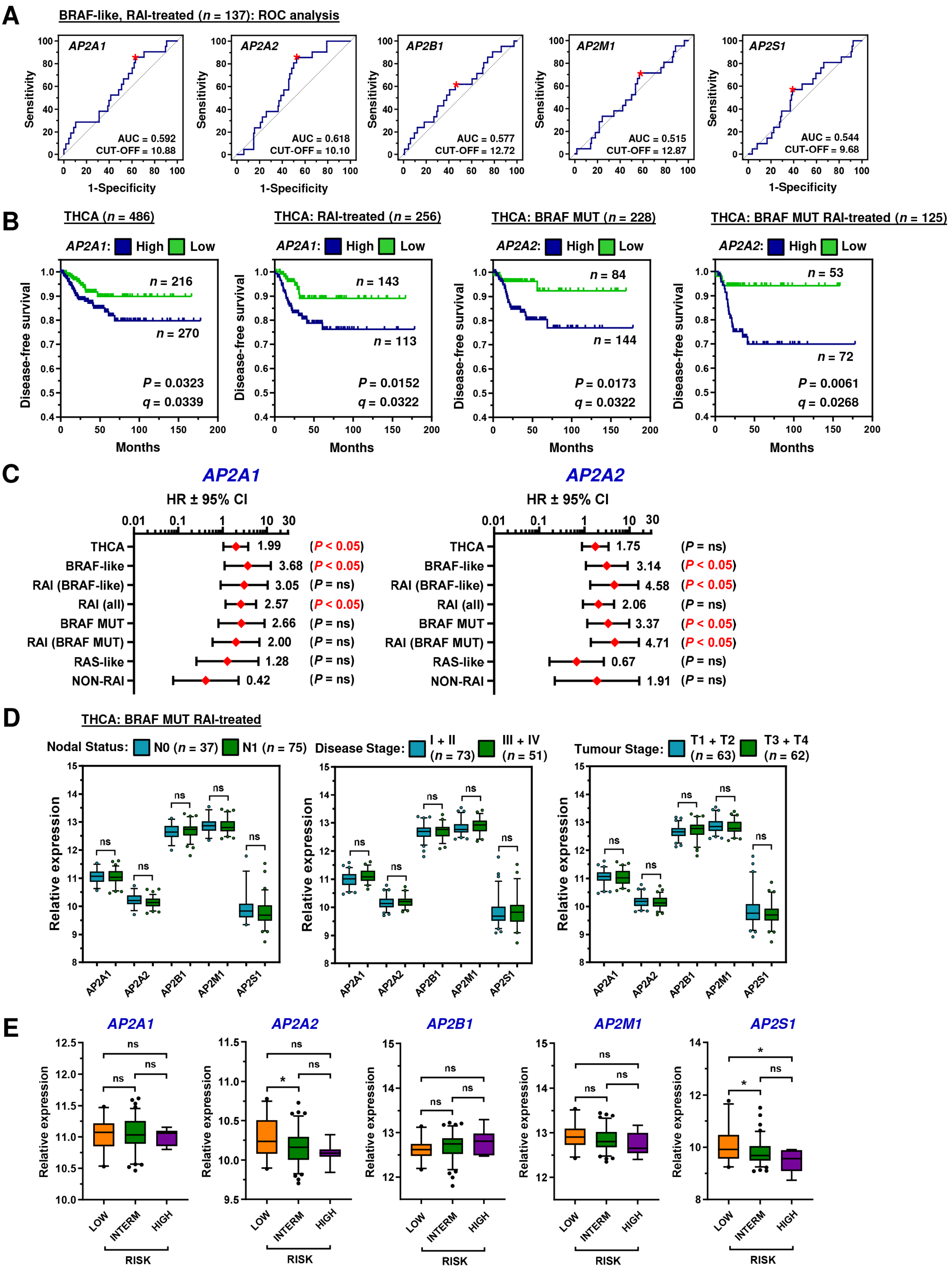

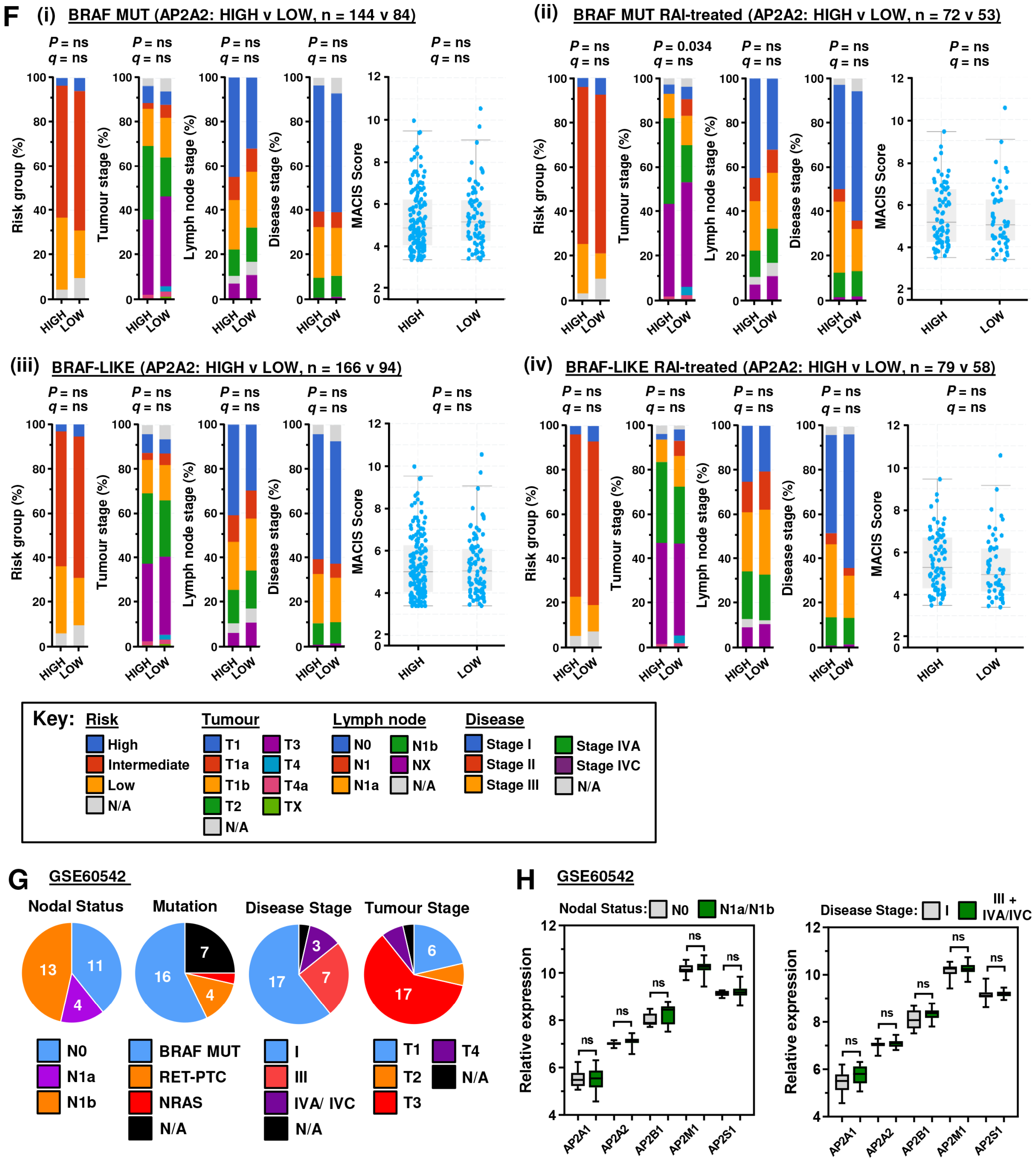

Figure S5. High AP2A1 and AP2A2 expression associated with increased risk of recurrence. **A,** Representative ROC curves of AP2 genes in the BRAF-like, RAI-treated THCA cohort (n = 137). **B,** Representative Kaplan-Meier analysis of DFS for indicated THCA TCGA cohorts stratified on high versus low tumoral expression for either *AP2A1* or *AP2A2*; log-rank test. Number (n) of patients per expression sub-group (high/low), P-values and q-values are shown. **C**, Hazard ratio (HR) ± 95% CI for patients stratified on AP2A1 and AP2A2 expression in THCA with the indicated treatment and genetic signature or alteration. **D,** Box and whisker plots showing expression ($\log_2$) of indicated AP2 genes in the BRAF MUT, RAI-treated THCA cohort stratified on nodal status (*left*), disease stage (*middle*) and tumour stage (*right*); P-values determined by Mann-Whitney test and adjusted using the Benjamini-Hochberg FDR correction procedure (ns, not significant). **E,** Same as **D** but showing expression (log2) of indicated AP2 genes in the BRAF MUT, RAI-treated THCA cohort stratified according to risk criteria [i.e. low, intermediate (INTERM) and high]. (ns, not significant; $^*P < 0.05$). **F,** Stacked bar charts showing clinical characteristics of THCA cohorts (i) BRAF MUT (n = 228), (ii) BRAF

MUT RAI-treated (n = 125), (iii) BRAF-like (n = 260) and (iv) BRAF-like RAI-treated (n = 137) stratified by AP2A2 expression (high versus low). Cut-off expression value used was 10.10. Clinical staging attributes include risk group, tumour staging, lymph node staging, disease staging and MACIS score. *P*-values derived using Chi-Squared test and adjusted using the Benjamini-Hochberg FDR correction procedure. **G,** Pie charts showing cancer staging and tumour mutational characteristics of patients in the GSE60542 cohort (*n* = 28). **H,** Box and whisker plots showing expression (log_2) of indicated AP2 genes in the GSE60542 cohort stratified on nodal status (*left*) and disease stage (*right*); *P*-values determined by Mann-Whitney test and adjusted using the Benjamini-Hochberg FDR correction procedure (ns, not significant).
