# Supplementary material for: Combined Vorinostat and Chloroquine Inhibit Sodium Iodide Symporter Endocytosis and Enhance Radionuclide Uptake In Vivo
Source: Clin Cancer Res. Author manuscript; Available in PMC 2024 Apr 1. (PMC7615786; doi:10.1158/1078-0432.CCR-23-2043)
Supplement: Supplementary Figure S6 [file EMS190879-supplement-Supplementary_Figure_S6.pdf]

SUPPLEMENTARY FIGURE S6

A BRAF-like (*n* = 260)

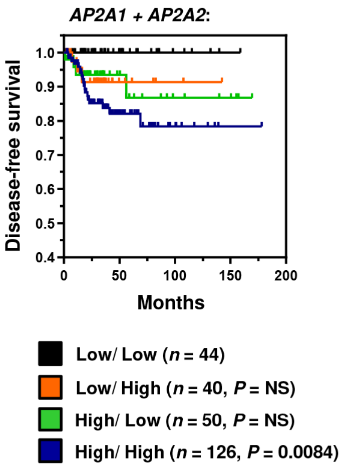

B THCA: BRAF-like RAI-treated (*n* = 137)

Gene #1	Gene #2	Expression Group				Disease-Free Survival (L/L vs H/H)		Frequency of Recurrence (L/L vs H/H)	
		Low/Low		High/High					
		N_{tot}	N_{rec}	N_{tot}	N_{rec}	*P*	*q*	*P*	*q*
AP2A1	*AP2A2*	29	0	62	15	0.006	0.038	0.002	0.013
AP2A1	*AP2B1*	31	1	51	11	0.034	0.071	0.026	0.055
AP2A1	*AP2M1*	34	2	42	5	0.411	0.432	0.450	0.473
AP2A1	*AP2S1*	23	2	55	8	0.619	0.557	0.714	0.643
AP2A2	*AP2B1*	31	2	39	12	0.020	0.063	0.015	0.047
AP2A2	*AP2M1*	42	1	38	4	0.151	0.238	0.185	0.291
AP2A2	*AP2S1*	32	2	54	8	0.260	0.328	0.310	0.391

C

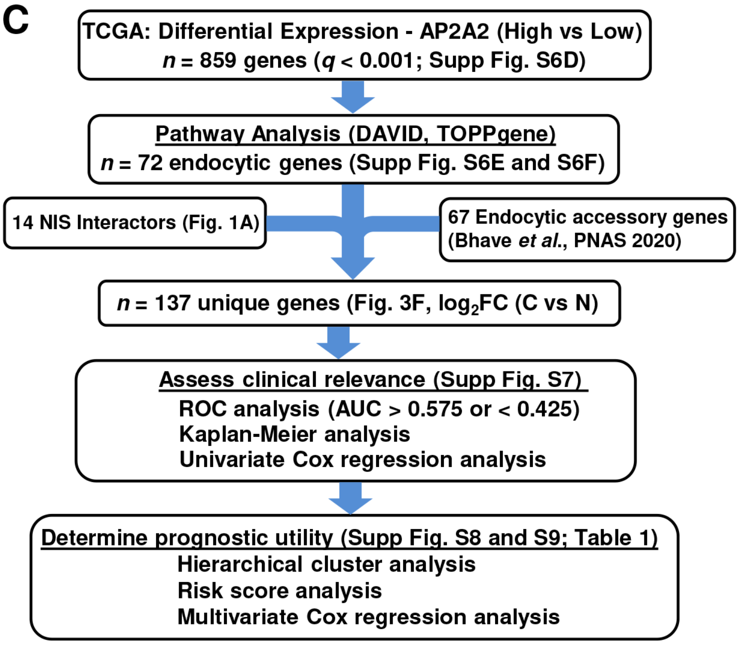

D THCA: BRAF-like RAI-treated (*n* = 137)

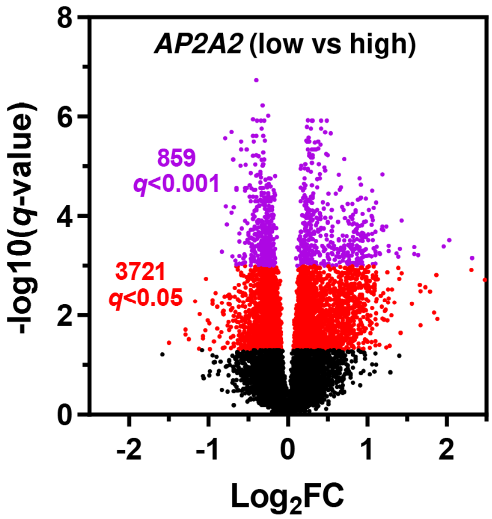

E DAVID: KEGG_PATHWAY

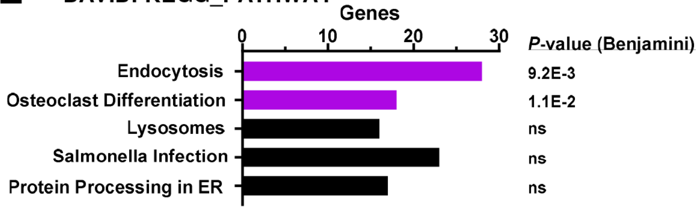

DAVID: BIOLOGICAL PROCESS

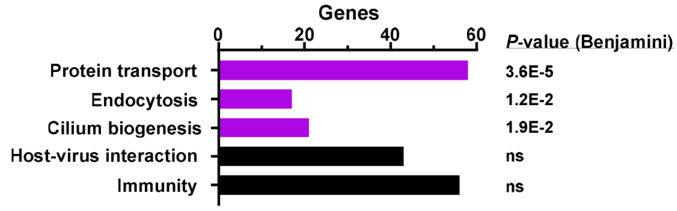

F ToppGene: PATHWAY

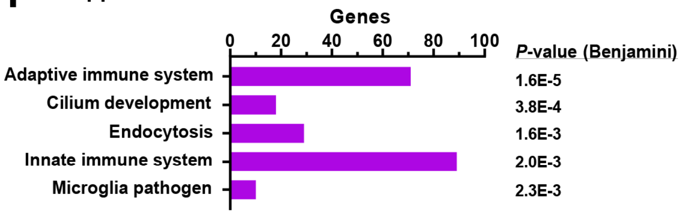

ToppGene: GO BIOLOGICAL PROCESS

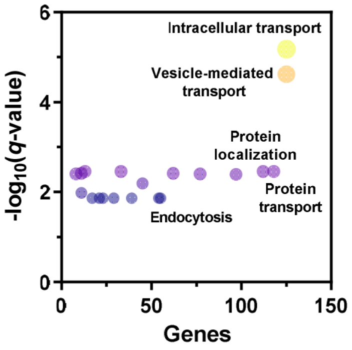

Figure S6. Identification of endocytic gene biomarkers for recurrence. **A,** Representative Kaplan-Meier analysis of DFS for the BRAF-like THCA cohort stratified on high vs low tumoral expression of *AP2A1* and *AP2A2*; log-rank test. Number (*n*) of patients per sub-group (high/low) and *P*-values are shown. **B,** Kaplan-Meier analysis of the BRAF-like, RAI-treated THCA cohort stratified on high (H/H) versus low (L/L) tumoral expression of either *AP2A1* or *AP2A2* combined with other AP2 genes; log-rank test. Number (N_{tot}) of patients and recurrent cases (N_{rec}) in each stratified group are shown, as well as *P*- and *q*-values. Fisher's exact test used to determine significance for incidence of recurrence between groups with high (H/H) vs low (L/L) tumoural gene expression. **C,** Bioinformatic pipeline used to filter and identify potential clinical biomarkers of PTC recurrence. **D,** Volcano plot comparing $\log_2$FC with *q*-value (-log base 10) for the BRAF-like, RAI-treated THCA dataset [high vs low AP2A2 expression; $n = 137$]. Top 859 genes ($q < 0.001$; purple spots) were filtered based on functional classification. **E,** DAVID functional classification of top 859 differentially expressed genes as described in **D**. KEGG pathway (*upper*) and biological process (*lower*) categories of greatest significance ($P < 0.05$ or lower, purple bars; ns, not significant, black bars) and number of genes per category are indicated. **F,** Same as **E** but ToppGene used to classify the top 859 differentially expressed genes ($q < 0.001$).
