# Supplementary material for: Combined Vorinostat and Chloroquine Inhibit Sodium Iodide Symporter Endocytosis and Enhance Radionuclide Uptake In Vivo
Source: Clin Cancer Res. Author manuscript; Available in PMC 2024 Apr 1. (PMC7615786; doi:10.1158/1078-0432.CCR-23-2043)
Supplement: Supplementary Figure S7 [file EMS190879-supplement-Supplementary_Figure_S7.pdf]

SUPPLEMENTARY FIGURE S7

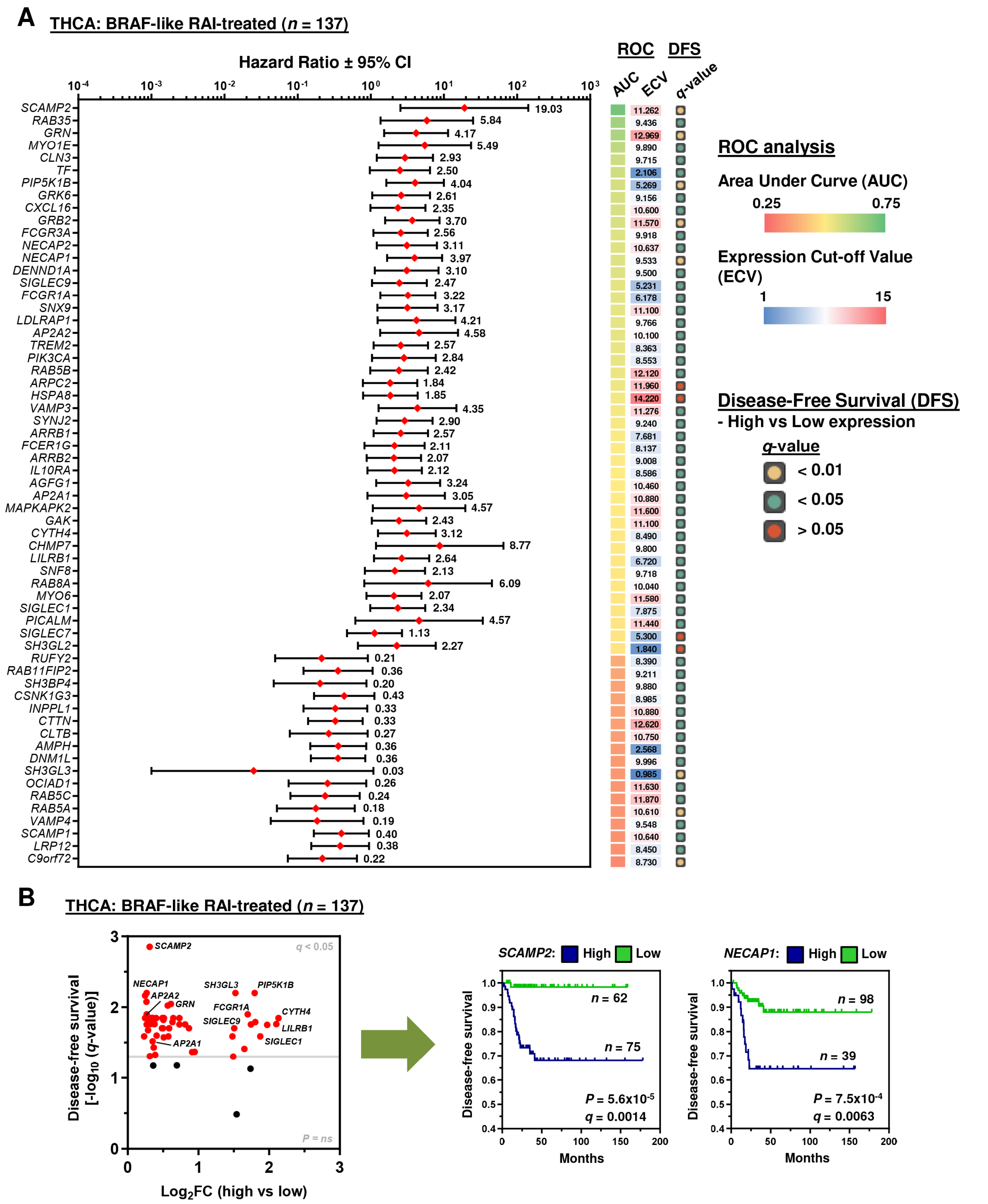

Figure S7. Comparison of the clinical relevance of filtered endocytic genes. **A**, Univariate Cox regression and ROC analysis of endocytic genes in BRAF-like, RAI treated THCA (n = 137) with AUC > 0.575 or < 0.425 (n = 61 out of 137 filtered endocytic genes fit this criteria). AUC, area under curve; ECV; expression cut-off value used for patient cohort stratification; DFS, disease-free survival; CI, confidence interval. **B,** Volcano plot illustrating $\log_2$FC in BRAF-like, RAI-treated THCA cohort (high vs low expression) compared to q-value (-log base 10) of recurrence for 61 endocytic genes (q < 0.05). (*right*) Representative Kaplan-Meier analysis of DFS for the BRAF-like, RAI treated THCA cohorts stratified on high vs low tumoural expression of *SCAMP2* and *NECAP1*; log-rank test. Number (n) of patients per sub-group (high/low), as well as P- and q-values are shown.
