# Supplementary material for: Combined Vorinostat and Chloroquine Inhibit Sodium Iodide Symporter Endocytosis and Enhance Radionuclide Uptake In Vivo
Source: Clin Cancer Res. Author manuscript; Available in PMC 2024 Apr 1. (PMC7615786; doi:10.1158/1078-0432.CCR-23-2043)
Supplement: Supplementary Figure S8 [file EMS190879-supplement-Supplementary_Figure_S8.pdf]

SUPPLEMENTARY FIGURE S8

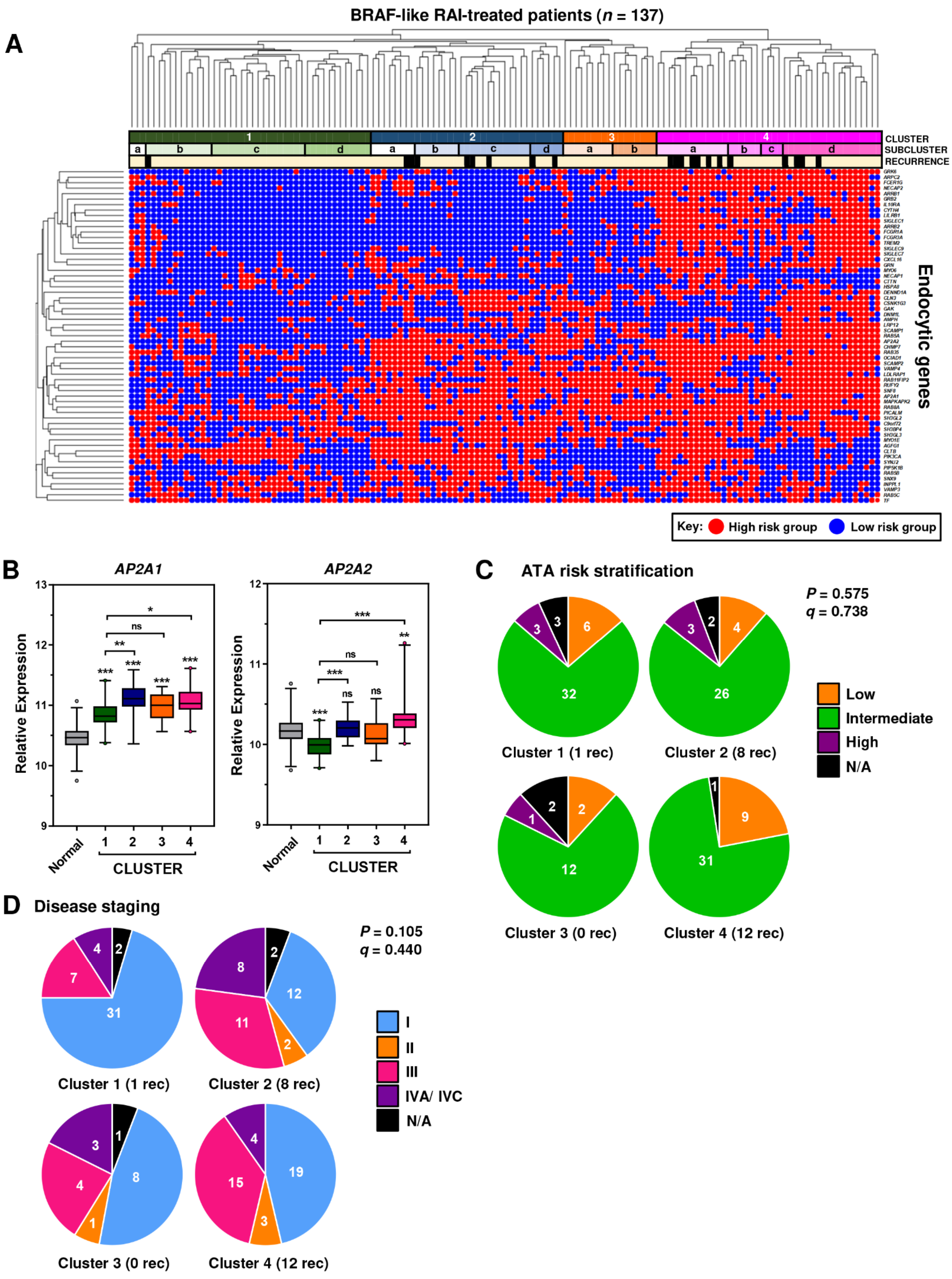

Figure S8. Greater endocytic gene dysregulation and higher AP2α expression in recurrent PTC. **A,** Hierarchical cluster analysis of the BRAF-like, RAI-treated THCA cohort (*n* = 137) based on endocytic genes (*n* = 61) stratified into high and low risk expression groups. ROC analysis was used to determine optimal expression cut-off values for stratifying patients into high and low risk groups. Patients were divided into 4 major clusters (1 to 4) and 14 subclusters (1a to 4d). Patients with recurrent disease are indicated (black squares). **B,** Box and whisker plots showing expression (log_2) of *AP2A1* (*left*) and *AP2A2* (*right*) in the BRAF-like, RAI-treated THCA cohort stratified into patient clusters 1 to 4 vs normal. Data presented as mean ± S.E.M., one-way ANOVA followed by Kruskal-Wallis test (ns, not significant; *P < 0.05; **P < 0.01; ***P < 0.001). **C,** Pie charts showing the ATA risk stratification characteristics of BRAF-like, RAI-treated patients subdivided into 4 clusters. Rec- number of recurrences. **D,** Same as **C** but showing disease staging characteristics as indicated. P-values derived using Chi-Squared test and adjusted using the Benjamini-Hochberg FDR correction procedure.
