# Supplementary material for: Combined Vorinostat and Chloroquine Inhibit Sodium Iodide Symporter Endocytosis and Enhance Radionuclide Uptake In Vivo
Source: Clin Cancer Res. Author manuscript; Available in PMC 2024 Apr 1. (PMC7615786; doi:10.1158/1078-0432.CCR-23-2043)
Supplement: Supplementary Figure S9 [file EMS190879-supplement-Supplementary_Figure_S9.pdf]

A THCA: BRAF-like RAI-treated (*n* = 137)

Gene Classifier	Cut-Off value	Sensitivity%	Specificity%	ROC		DFS	HR	95% CI
				AUC	q-value	q-value		
10	8.756	71.43	81.03	0.8206	<0.0001	<0.0001	9.131	3.527, 23.639
20	25.22	90.48	89.66	0.9179	<0.0001	<0.0001	52.782	12.187, 228.605
30	27.62	85.71	94.83	0.9319	<0.0001	<0.0001	57.265	16.489, 198.873
40	37.82	95.24	81.03	0.9397	<0.0001	<0.0001	61.305	8.212, 457.659

Classifier	Genes
10	*RAB35, SCAMP2, GRK6, CXCL16, AP2A2, NECAP1, CTTN, INPPL1, AP2A1, RAB5B*
20	*& CHMP7, CLTB, IL10RA, HSPA8, ARRB2, GRN, DENND1A, SYNJ2, FCGR1A, SH3GL3*
30	*& FCGR3A, NECAP2, VAMP4, ARRB1, AGFG1, LILRB1, MYO6, OCIAD1, CYTH4, MYO1E*
40	*& TREM2, SIGLEC9, SIGLEC1, ARPC2, FCER1G, GRB2, SH3GL2, PIP5K1B, PIK3CA, SIGLEC7*

B

Gene Classifier	Validation datasets			
	BRAF MUT n = 228	RAI-treated n = 256	BRAF-like n = 260	THCA n = 486
	AUC, q-value	AUC, q-value	AUC, q-value	AUC, q-value
10	0.6845, 0.0023	0.6814, 0.0010	0.7229, 0.0003	0.5854, ns
20	0.7669, < 0.0001	0.7366, < 0.0001	0.8092, < 0.0001	0.6336, 0.0030
30	0.7664, < 0.0001	0.7447, < 0.0001	0.8144, < 0.0001	0.6421, 0.0020
40	0.7675, < 0.0001	0.7508, < 0.0001	0.8116, < 0.0001	0.6373, 0.0025

C

Patient Cohort	N_{tot}	N_{rec}	30 Endocytic Gene Classifier				40 Endocytic Gene Classifier			
			DFS		Univariate		DFS		Univariate	
			P-value	*q*-value	HR (95% CI)	*P*-value	*P*-value	*q*-value	HR (95% CI)	*P*-value
THCA	486	46	4.14×10^{-11}	8.28×10^{-11}	5.717 (3.187-10.257)	5.04×10^{-9}	8.99×10^{-7}	1.25×10^{-6}	3.861 (2.161-6.897)	5.05×10^{-6}
BRAF-like	260	26	6.91×10^{-17}	3.11×10^{-16}	15.501 (6.604-36.383)	3.05×10^{-10}	4.13×10^{-10}	7.43×10^{-10}	10.631 (4.241-26.650)	4.63×10^{-7}
BRAF-like, RAI-treated	137	21	6.10×10^{-28}	1.10×10^{-26}	57.265 (16.489-198.873)	1.86×10^{-10}	1.03×10^{-13}	3.71×10^{-13}	61.305 (8.212-457.659)	5.99×10^{-5}
RAI-treated	256	34	2.01×10^{-21}	1.81×10^{-20}	13.680 (6.798-27.530)	2.28×10^{-13}	3.86×10^{-12}	9.93×10^{-12}	8.421 (4.098-17.305)	6.69×10^{-9}
BRAF MUT	228	27	5.51×10^{-13}	1.65×10^{-12}	10.953 (4.871-24.628)	7.06×10^{-9}	4.39×10^{-8}	6.59×10^{-8}	7.767 (3.270-18.452)	3.42×10^{-6}
BRAF MUT, RAI-treated	125	21	2.48×10^{-20}	1.49×10^{-19}	38.250 (11.139-131.341)	7.06×10^{-9}	8.75×10^{-12}	1.97×10^{-11}	51.72 (6.930-385.975)	1.19×10^{-4}
RAS-like	115	8	0.504	0.504	ns	0.661	0.376	0.398	ns	0.565
Non RAI-treated	167	6	0.262	0.295	ns	0.480	0.146	0.175	ns	0.379

D 30 Endocytic Gene Classifier

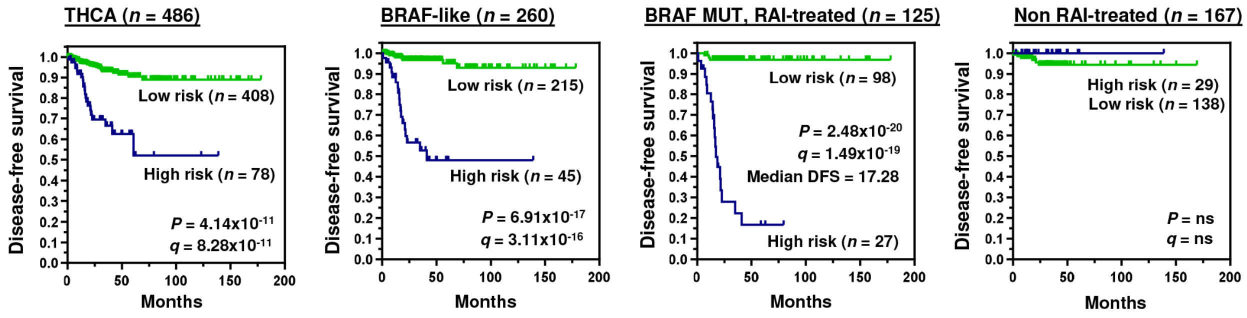

Figure S9. Construction and validation of endocytic multigene classifiers for PTC recurrence. **A,** Prognostic profile of endocytic risk score multigene classifiers (*n* = 10 – 40 genes). Estimations of sensitivity and specificity (%) for the BRAF-like, RAI-treated THCA cohort (*n* = 137) are given, as well as expression cut-off values for stratification. AUC, area under curve; DFS, disease-free survival; HR, hazard ratio; CI, confidence interval. (*lower*) Genes included in the various classifiers are indicated. Gene order based on consolidation of the 40 gene risk score, in which genes were ranked on the relative proportion of their contribution towards the overall risk score value. **B,** ROC analysis (AUC) of different gene classifiers (*n* = 10 - 40) in validation larger THCA datasets as indicated. Red box indicates highest AUC value for each THCA cohort. **C,** Comparison of Kaplan-Meier and univariate Cox regression analysis in different THCA cohorts using either a 30- or 40 endocytic gene risk score classifier. Number of patients (N_{tot}) and recurrent cases (N_{rec}) in each patient cohort are given (ns, not significant). **D,** Representative Kaplan-Meier analysis of DFS for the THCA (*n* = 486), BRAF-like (*n* = 260), BRAF MUT RAI-treated (*n* = 125) and Non RAI-treated (*n* = 167) THCA cohorts stratified using the 30 endocytic gene classifier; log-rank test. Number (n) of patients per sub-group (high/low), as well as *P*- and *q*-values are shown.
