# Supplementary material for: Combined Vorinostat and Chloroquine Inhibit Sodium Iodide Symporter Endocytosis and Enhance Radionuclide Uptake In Vivo
Source: Clin Cancer Res. Author manuscript; Available in PMC 2024 Apr 1. (PMC7615786; doi:10.1158/1078-0432.CCR-23-2043)
Supplement: Supplementary Figure S10 [file EMS190879-supplement-Supplementary_Figure_S10.pdf]

SUPPLEMENTARY FIGURE S10

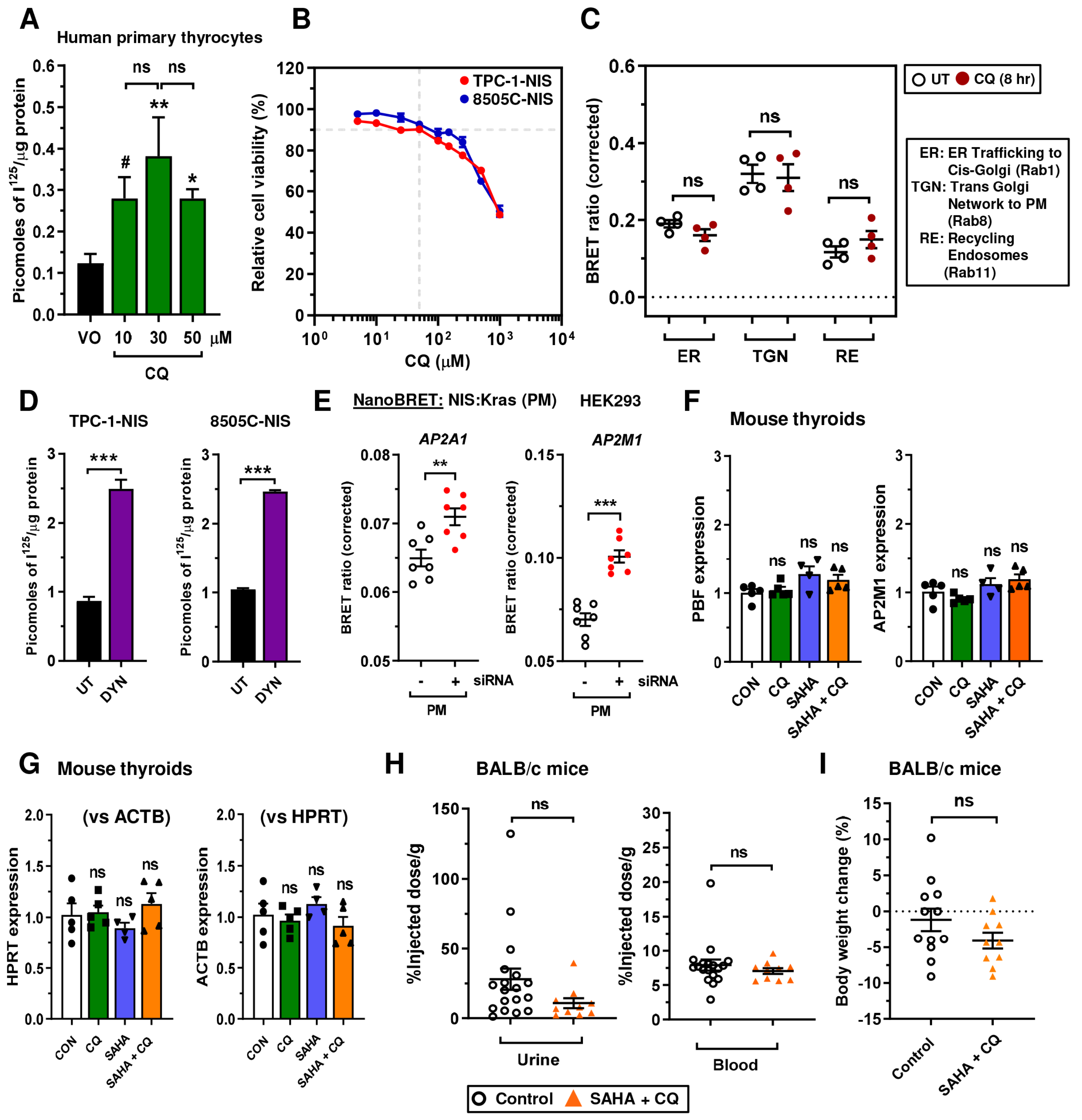

Figure S10. Biological effects and validation of targeting endocytic pathways. **A,** Radioiodide uptake of human primary thyrocytes treated with CQ at the indicated dose vs vehicle only (VO). Data presented as mean ± S.E.M., one-way ANOVA followed by Tukey's multiple comparison test (**P* < 0.05; ***P* < 0.01) or unpaired two-tailed t-test (#*P* < 0.05). **B,** Cell viability of TPC-1-NIS and 8505C-NIS cells treated with CQ for 8 hours at the indicated dose (n = 3). CQ dose of 50 μM is highlighted (dashed grey lines). **C,** Profiling subcellular changes of NIS using the NanoBRET assay in CQ-treated HeLa cells. HeLa cells were transiently transfected with NIS tagged with NLuc, and the subcellular markers Rab1 (ER trafficking to cis-golgi), Rab8 (Trans golgi network to PM) or Rab11 (Recycling endosomes) tagged with Venus. **D,** RAI uptake in TPC-1-NIS and 8505C-NIS cells following Dynasore (DYN) treatment. **E,** NanoBRET evaluation of NIS PM localisation in HEK293 cells transfected with siRNA specific for indicated AP2 genes. **F,** PBF and AP2M1 mRNA levels in thyroid glands dissected from WT BALB/c mice administered with CQ and SAHA either alone or in combination. **G,** Same as (**F**) but with HPRT and ACTB mRNA levels. **H,** Technetium-99m pertechnetate levels in urine and blood harvested from WT BALB/c mice administered with CQ and SAHA either alone or in combination. **I,** Body weight change (%) in WT BALB/c mice administered with a combination of CQ and SAHA (n = 10) versus controls (n = 12). Data presented as mean ± S.E.M., ns, not significant; **P* < 0.05; ****P* < 0.001.
