# Supplementary material for: Combined Vorinostat and Chloroquine Inhibit Sodium Iodide Symporter Endocytosis and Enhance Radionuclide Uptake In Vivo
Source: Clin Cancer Res. Author manuscript; Available in PMC 2024 Apr 1. (PMC7615786; doi:10.1158/1078-0432.CCR-23-2043)
Supplement: Supplementary Figure S11 [file EMS190879-supplement-Supplementary_Figure_S11.pdf]

SUPPLEMENTARY FIGURE S11

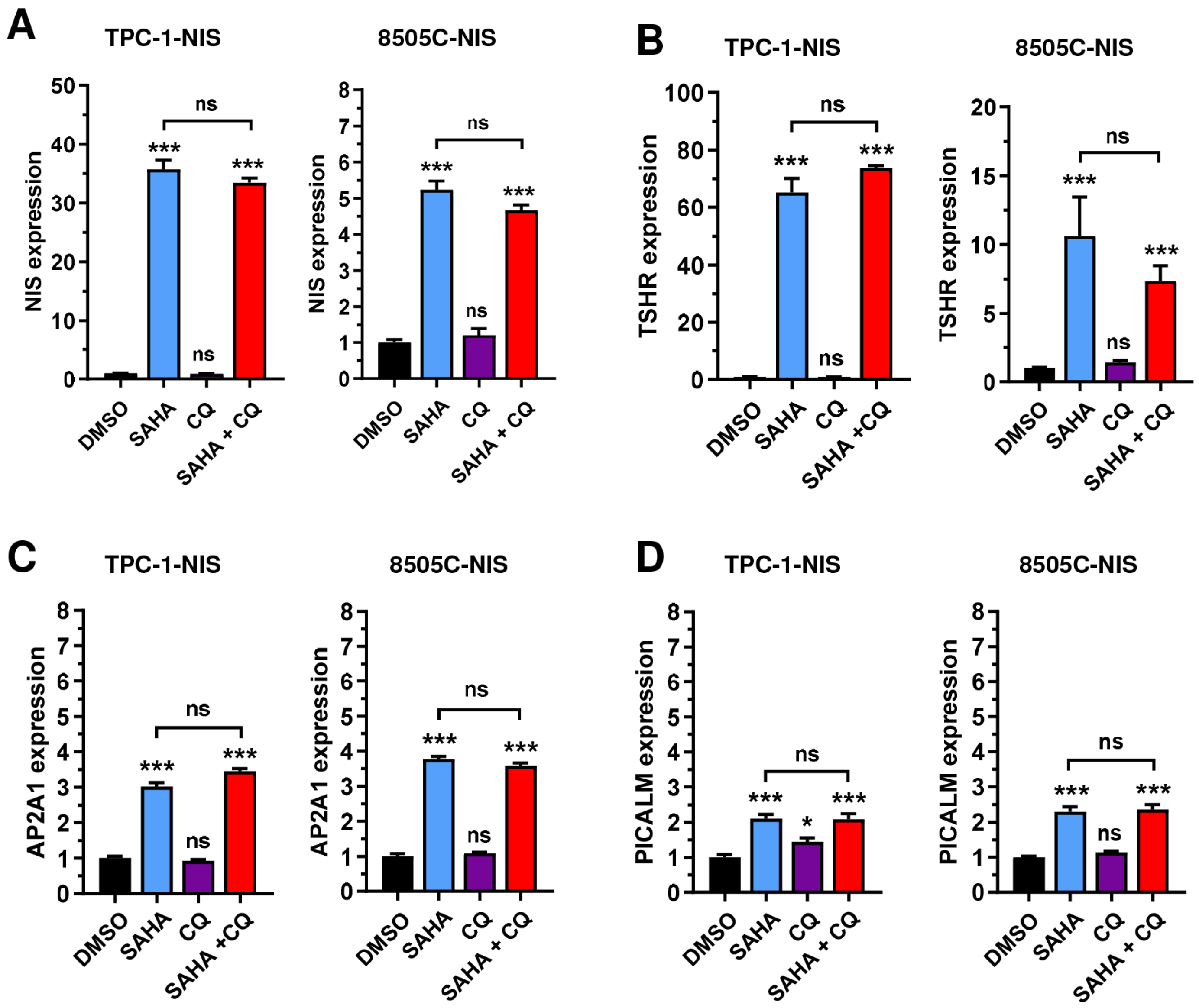

Figure S11. Transcriptional drug responses in thyroid cancer cells. **A-D,** Relative NIS (**A**), TSHR (**B**), AP2A1 (**C**) and PICALM (**D**) mRNA levels in TPC-1-NIS and 8505C-NIS cells treated with CQ and SAHA either alone or in combination. Data presented as mean ± S.E.M., one-way ANOVA followed by Tukey's post hoc test (ns, not significant; **P* < 0.05; ****P* < 0.001).
