# Supplementary material for: Combined Vorinostat and Chloroquine Inhibit Sodium Iodide Symporter Endocytosis and Enhance Radionuclide Uptake In Vivo
Source: Clin Cancer Res. Author manuscript; Available in PMC 2024 Apr 1. (PMC7615786; doi:10.1158/1078-0432.CCR-23-2043)
Supplement: Supplementary Information. This file includes Supplementary Materials and Methods [file EMS190879-supplement-Supplementary_Information__This_file_includes_Supplementary_Materials_and_Methods.docx]

**Supplementary Information**

**Combined Vorinostat and Chloroquine Inhibit Sodium Iodide Symporter Endocytosis and Enhance Radionuclide Uptake In Vivo**

Martin L. Read^1ǂ^, Katie Brookes^1ǂ^, Ling Zha^1ǂ^, Selvambigai Manivannan^1^, Jana Kim^2^, Merve Kocbiyik^1^, Alice Fletcher^1^, Caroline M. Gorvin^1^, George Firth^2^, Gilbert O. Fruhwirth^3^, Juan P. Nicola^4^, Sissy Jhiang^5^, Matthew D. Ringel^5^, Moray J. Campbell^6^, Kavitha Sunassee^2^, Philip J. Blower^2^, Kristien Boelaert^7^, Hannah R. Nieto^1^, Vicki E. Smith^1^, Christopher J. McCabe^1*^

^1^Institute of Metabolism and Systems Research (IMSR), and Centre of Endocrinology, Diabetes and Metabolism (CEDAM), University of Birmingham, Birmingham, UK

^2^School of Biomedical Engineering & Imaging Sciences, King’s College London, London, UK

^3^Comprehensive Cancer Centre, School of Cancer and Pharmaceutical Sciences, King's College London, Guy's Campus, London, UK

^4^Departamento de Bioquímica Clínica (CIBICI-CONICET), Facultad de Ciencias Químicas, Universidad Nacional de Córdoba, Córdoba, Argentina

^5^Divison of Endocrinology, Diabetes, and Metabolism and Cancer Biology Program, The Ohio State University College of Medicine and Comprehensive Cancer Center, Columbus, Ohio, USA

^6^Department of Pharmaceutics and Pharmaceutical Chemistry, College of Pharmacy at The Ohio State University, Columbus, Ohio, USA

^7^Institute of Applied Health Research, University of Birmingham, Birmingham, UK

^ǂ^Authors contributed equally.

^*^**Corresponding author**: Professor Christopher J. McCabe, Institute of Metabolism and Systems Research, Birmingham Health Partners, College of Medical and Dental Sciences, University of Birmingham, Birmingham, B15 2TH, UK. Email: [mccabcjz@bham.ac.uk](mailto:mccabcjz@bham.ac.uk); Tel.: +44 (0) 121 415 8713.

**This file includes:**

Supplementary Materials and Methods

**Supplementary Materials and Methods**

**Key resources**

| **REAGENT** | **SOURCE** | **IDENTIFIER** |
| --- | --- | --- |
| **Antibodies** |  |  |
| Rabbit polyclonal anti-AP2A1 | Signalway | Cat# 43215, RRID: AB_3073647 |
| Rabbit polyclonal anti-AP2M1 | Novus Biologicals | Cat# NBP1-32272, RRID: AB_2056484 |
| Rabbit polyclonal anti-NIS | Proteintech | Cat# 24324-1-AP, RRID: AB_2879495 |
| Rabbit monoclonal anti-PICALM | Cell Signaling Technology | Cat# 26765S, RRID: AB_3073648 |
| Rabbit polyclonal anti-Na,K-ATPase | Cell Signaling Technology | Cat# 3010, RRID: AB_2060983 |
| Mouse monoclonal anti-HA | BioLegend | Cat# 901502, RRID: AB_2565006 |
| Mouse monoclonal anti-β-actin | Sigma-Aldrich | Cat# A1978, RRID: AB_476692 |
| Goat polyclonal anti-rabbit immunoglobulins/ HRP | Agilent Technologies | Cat# P0448, RRID:AB_2617138 |
| Rabbit polyclonal anti-mouse immunoglobulins/ HRP | Agilent Technologies | Cat# P0260, RRID:AB_2636929 |
| Goat anti-Rabbit IgG secondary antibody, Alexa Fluor™ 555 | ThermoFisher Scientific | Cat# A-21428, RRID:AB_2535849 |
| Goat anti-Mouse IgG secondary antibody, Alexa Fluor™ 488 | ThermoFisher Scientific | Cat# A28175, RRID:AB_2536161 |
| **Bacterial and Virus Strains** |  |  |
| Subcloning efficiency^TM^ DH5α competent cells | ThermoFisher Scientific | Cat# 18265017 |
| **Biological Samples** |  |  |
| Human thyroid tissue | Queen Elizabeth Hospital, Birmingham, UK | N/A |
| **Oligonucleotides** |  |  |
| AAK1 TaqMan® Gene Expression Assay (human) | ThermoFisher Scientific | Hs00208618_m1 |
| AP2A1 TaqMan® Gene Expression Assay (human) | ThermoFisher Scientific | Hs00900330_m1 |
| AP2M1 TaqMan® Gene Expression Assay (human) | ThermoFisher Scientific | Hs01037584_m1 |
| AP2S1 TaqMan® Gene Expression Assay (human) | ThermoFisher Scientific | Hs00900330_m1 |
| PICALM TaqMan® Gene Expression Assay (human) | ThermoFisher Scientific | Hs00200318_m1 |
| PPIA TaqMan® Gene Expression Assay (human) | ThermoFisher Scientific | Hs04194521_s1 |
| SLC5A5 TaqMan® Gene Expression Assay (human) | ThermoFisher Scientific | Hs00950358_m1 |
| TSHR TaqMan® Gene Expression Assay (human) | ThermoFisher Scientific | Hs01053846_m1 |
| 18S TaqMan® Gene Expression Assay (human) | ThermoFisher Scientific | Hs03003631_g1 |
| ACTB TaqMan® Gene Expression Assay (mouse) | ThermoFisher Scientific | Mm01205647_g1 |
| AP2A1 TaqMan® Gene Expression Assay (mouse) | ThermoFisher Scientific | Mm00475919_m1 |
| AP2M1 TaqMan® Gene Expression Assay (mouse) | ThermoFisher Scientific | Mm05884025_g1 |
| HPRT TaqMan® Gene Expression Assay (mouse) | ThermoFisher Scientific | Mm00446968_m1 |
| NKX2-1 TaqMan® Gene Expression Assay (mouse) | ThermoFisher Scientific | Mm07296387_g1 |
| PAX8 TaqMan® Gene Expression Assay (mouse) | ThermoFisher Scientific | Mm00440623_m1 |
| PICALM TaqMan® Gene Expression Assay (mouse) | ThermoFisher Scientific | Mm00525455_m1 |
| PTTG1IP TaqMan® Gene Expression Assay (mouse) | ThermoFisher Scientific | Mm00521473_m1 |
| SLC5A5 TaqMan® Gene Expression Assay (mouse) | ThermoFisher Scientific | Mm01351811_m1 |
| TSHR TaqMan® Gene Expression Assay (mouse) | ThermoFisher Scientific | Mm00442027_m1 |
| ON-TARGETplus Human AAK1 siRNA | Horizon Discovery | Cat# L-005300-02-0005 |
| ON-TARGETplus Human AP2A1 siRNA | Horizon Discovery | Cat# L-012492-00-0005 |
| ON-TARGETplus Human AP2A2 siRNA | Horizon Discovery | Cat# L-012812-00-0005 |
| ON-TARGETplus Human AP2M1 siRNA | Horizon Discovery | Cat# L-008170-00-0005 |
| ON-TARGETplus Human AP2S1 siRNA | Horizon Discovery | Cat# L-011833-01-0005 |
| ON-TARGETplus Non-targeting Pool siRNA | Horizon Discovery | Cat# D-001810-10-05 |
| LgBiT (PCR primers; N-terminal)  5’-GCCAAGCTTACCATGGTCTTCACACTCGAA-3’  5’-GGCGGTACCACTGTTGATGGTTACTCG-3’ | Sigma-Aldrich | Custom synthesis |
| LgBiT (PCR primers; C-terminal)  5’-GCCTCTAGAGTCTTCACACTCGAAGAT-3’  5’-GGCGGCGGGCCCTTAGCTGTTGATGGTTAC-3’ | Sigma-Aldrich | Custom synthesis |
| AP2S1 (PCR primers; N-terminal tag with LgBiT)  5’-GCCGGTACCATCCGCTTTATCCTCATC-3’  5’-GCCTCTAGATCACTCCAGGGACTGTAG-3’ | Sigma-Aldrich | Custom synthesis |
| AP2S1 (PCR primers; C-terminal tag with LgBiT)  5’-GCCGGTACCACCATGATCCGCTTTATCCTC-3’  5’-GCCTCTAGACTCCAGGGACTGTAGCAT-3’ | Sigma-Aldrich | Custom synthesis |
| AP2S1 (PCR primers; N-terminal tag with SmBiT)  5’-GCCGGTACCACCATGGTGACCGGCTACCGGCT  GTTCGAGGAGATTCTCATCCGCTTTATCCTCA-3’  5’-GCCGCGCCGCCGCCTCTAGATCACTCCAGGGA  CTGT-3’ | Sigma-Aldrich | Custom synthesis |
| AP2S1 (PCR primers; C-terminal tag with SmBiT)  5’-GCCGCCGCCGCCGGTACCACCATGATCCGCT  TTATCC-3’  5’-GCCTCTAGATTACAGAATCTCCTCGAACAGCC  GGTAGCCGGTCACCTCCAGGGACTGTAGC-3’ | Sigma-Aldrich | Custom synthesis |
| PBF (PCR primers; N-terminal tag with LgBiT)  5’- GCCGGTACCGCGCCCGGAGTGGCCCG-3’  5’- GCCTCTAGATTAGTTGTTTTCAAATCT-3’ | Sigma-Aldrich | Custom synthesis |
| PBF (PCR primers; C-terminal tag with LgBiT)  5’-GCCGGTACCACCATGGCGCCCGGAGTGGC-3’  5’-GCCTCTAGAGTTGTTTTCAAATCTAGC-3’ | Sigma-Aldrich | Custom synthesis |
| PBF (PCR primers; N-terminal tag with SmBiT)  5’-GCCGGTACCACCATGGTGACCGGCTACCGGCT  GTTCGAGGAGATTCTCGCGCCCGGAGTGGCCC-3’  5’-GCCGCGCCGCCGCCTCTAGATTAGTTGTTTTCA  AATC-3’ | Sigma-Aldrich | Custom synthesis |
| PBF (PCR primers; C-terminal tag with SmBiT)  5’-GCCGCCGCCGCCGGTACCACCATGGCGCCCG  GAGTG-3’  5’-GCCTCTAGAGAGAATCTCCTCGAACAGCCGGTA  GCCGGTCACGTTGTTTTCAAATCTAG-3’ | Sigma-Aldrich | Custom synthesis |
| NIS (mutagenesis primers; L562A and L563A)  5’-CCCCGGGAGCGGCGTGGTGGGAC-3’  5’-GTCCCACCACGCCGCTCCCGGGG-3’ | Sigma-Aldrich | Custom synthesis |
| NIS (mutagenesis primers; E578A and E579A)  5’-GGATGGCCACTGCTGCCTTGGGG-3’  5’-CCCCAAGGCAGCAGTGGCCATCC-3’ | Sigma-Aldrich | Custom synthesis |
| AP2S2 (mutagenesis primers; V88D)  5’-AGGCCATTCACAACTTCGACGAGGTCTTAAACG AATATTT-3’  5’-AAATATTCGTTTAAGACCTCGTCGAAGTTGTGAA  TGGCCT-3’ | Sigma-Aldrich | Custom synthesis |
| AP2S2 (mutagenesis primers; L103D)  5’-CAATGTCTGTGAACTGGACAGCGTGTTCAACTT CTACAAG-3’  5’-CTTGTAGAAGTTGAACACGCTGTCCAGTTCACA  GACATTG-3’ | Sigma-Aldrich | Custom synthesis |
| **Recombinant DNA** |  |  |
| pcDNA3.1(+) | ThermoFisher Scientific | Cat# V79020 |
| pcDNA3.1-NIS-HA | Smith VE et al., 2009 | Supp Ref (1) |
| pcDNA3.1-PBF-HA | Smith VE et al., 2009 | Supp Ref (1) |
| pcDNA3.1-NIS-SmBiT | Read ML et al., 2022 | Supp Ref (2) |
| pcDNA3.1-NIS-LgBiT | This paper | N/A |
| pcDNA3.1-PBF-SmBiT | This paper | N/A |
| pcDNA3.1-PBF-LgBiT | This paper | N/A |
| pcDNA3.1-LgBiT (N-terminal; HindIII/KpnI) | This paper | N/A |
| pcDNA3.1-LgBiT (C-terminal; XbaI/ApaI) | This paper | N/A |
| pcDNA3.1-AP2S1-LgBiT | This paper | N/A |
| pcDNA3.1-LgBiT-AP2S1 | This paper | N/A |
| pcDNA3.1-AP2S1-SmBiT | This paper | N/A |
| pcDNA3.1-SmBiT-AP2S1 | This paper | N/A |
| pcDNA3.1-NIS(L562A/L563A)-SmBiT | This paper | N/A |
| pcDNA3.1-NIS(E578A/E579A)-SmBiT | This paper | N/A |
| pcDNA3.1-LgBiT-AP2S1(V88D) | This paper | N/A |
| pcDNA3.1-LgBiT-AP2S1(L103S) | This paper | N/A |
| pcDNA3.1-NIS(L562A/L563A)-HA | This paper | N/A |
| pcDNA3.1-NIS(E578A/E579A)-HA | This paper | N/A |
| pcDNA3.1-AP2S1(V88D) | This paper | N/A |
| pcDNA3.1-AP2S1(L103S) | This paper | N/A |
| pcDNA3.1-Rab1-Venus | Kevin Pfleger’s lab | Supp Ref (3) |
| pcDNA3.1-Rab4-Venus | Kevin Pfleger’s lab | Supp Ref (3) |
| pcDNA3.1-Rab6-Venus | Kevin Pfleger’s lab | Supp Ref (3) |
| pcDNA3.1-Rab8-Venus | Kevin Pfleger’s lab | Supp Ref (3) |
| pcDNA3.1-Rab9-Venus | Kevin Pfleger’s lab | Supp Ref (3) |
| pcDNA3.1-Kras-Venus | Nevin Lambert’s lab | Supp Ref (4) |
| pcDNA3.1-Rab5-Venus | Nevin Lambert’s lab | Supp Ref (4) |
| pcDNA3.1-Rab7-Venus | Nevin Lambert’s lab | Supp Ref (4) |
| pcDNA3.1-Rab11-Venus | Nevin Lambert’s lab | Supp Ref (4) |
| pcDNA3.1-NIS-Nluc | ThermoFisher Scientific | Custom synthesis |
| pCMV3-AP2S1-untagged | Sino Biological Europe GmbH | Cat# HG12478-UT |
| **Experimental Models: Cell Lines** |  |  |
| HEK293 | ECACC | Cat# 85120602, RRID: CVCL_0045 |
| HeLa | ECACC | Cat# 93021013, RRID: CVCL_0030 |
| TPC-1 | Rebecca Schweppe’s lab | RRID:CVCL_6298 |
| 8505C | DSMZ | Cat# ACC-219, RRID: CVCL_1054 |
| SW1736 | Rebecca Schweppe’s lab | RRID:CVCL_3883 |
| TPC-1-NIS | Read ML et al., 2022 | Supp Ref (2) |
| 8505C-NIS | This paper | N/A |
| **Experimental Models: Organisms/Strains** |  |  |
| BALB/cAnNCrl (BALB/c) | Charles River | RRID:IMSR_CRL:028 |
| **Deposited data** |  |  |
| Thyroid carcinoma (TCGA, Firehose Legacy) | Supp Ref (5, 6) | https:www.cbioportal.org/study/summary?id=thca_tcga  RRID:SCR_014555 |
| TCGA GDAC Firehose standard data – Thyroid Carcinoma (THCA) | Broad Institute of MIT and Harvard | https://doi.org/10.7908/C11G0KM9  http://firebrowse.org |
| TCGA THCA | Supp Ref (7) | https://portal.gdc.cancer.gov/projects/TCGA-THCA  RRID:SCR_014514 |
| GSE60542 | Supp Ref (8) | https://www.ncbi.nlm.nih.gov/geo/geo2r/?acc=GSE60542 |
| **Software** |  |  |
| GraphPad Prism Version 9.5 | Graphpad Software Inc | RRID:SCR_002798 |
| Excel 2016 | Microsoft | RRID:SCR_016137 |
| PowerPoint 2016 | Microsoft | RRID:SCR_023631 |
| EndNote 20 | Clarivate | RRID:SCR_014001 |
| SPSS Statistics Version 29 | IBM | RRID:SCR_002865 |
| GEO2R | National Center for Biotechnology Information | RRID:SCR_016569 |
| BioRender | http://biorender.com | RRID:SCR_018361 |
| ImageJ | National Institutes of Health | RRID:SCR_003070 |
| Morpheus | Broad Institute | RRID:SCR_017386 |
| DAVID Bioinformatics | National Institutes of Health | RRID:SCR_001881 |
| ToppGene Suite | Cincinnati Children's Hospital Medical Center | RRID:SCR_005726 |

**Construction of NanoBiT LgBiT- and SmBiT-tagged genes**

The LgBiT tag was amplified with Q5 DNA Polymerase (NEB) using either N- or C-terminal LgBiT primers (see Key resources; NanoBiT PPI starter system; N2014). Following gel extraction (QIAquick kit; Qiagen), PCR products were digested with HindIII and KpnI (Promega) for N-terminal tagging or with XbaI and ApaI (Promega) for C-terminal tagging, prior to ligation to pcDNA3.1(+) (ThermoFisher Scientific) linearised with the same pairs of restriction enzymes. Next, AP2S1 cDNA was amplified using specific primers for N- and C-terminal LgBiT tagging (see Key resources). PCR products were digested with KpnI and XbaI, and then ligated (T4 DNA ligase; NEB) into LgBiT-containing vectors [pcDNA3.1-LgBiT (N-terminal); pcDNA3.1-LgBiT (C-terminal)] linearised with the same restriction enzymes. AP2S1 and PBF cDNA were amplified with the SmBiT tag directly using appropriate PCR primers (see Key resources) and ligated into the empty pcDNA3.1(+) vector linearised with the same restriction enzymes.

**NanoBiT and NanoBRET live cell assays**

Cells were seeded in 6-well plates at a density of 3.5 x 10^5^ cells per well and transfected with 500 ng – 1 µg plasmid DNA. 24 hours post-transfection, cells were harvested and reseeded into white 96-well plates in phenol-red-free DMEM (Life Technologies). Furimazine (Promega) was added to each well in accordance with the manufacturer’s guidelines and readings taken at 120 second intervals for up to 40 minutes (PHERAstar FS microplate reader; BMG Labtech). In some experiments, cells were treated with CQ (8 hours) or DYN (24 hours) prior to addition of furimazine. NanoBRET signal was calculated using standard protocols by dividing the acceptor emission at 618 nm by the donor emission at 460 nm.

**Cell surface biotinylation assay (CSBA)**

The principle of the CBSA was to isolate PM proteins by biochemically labelling and separating proteins on the PM from those in intracellular compartments. An overview and description of the main steps involved are outlined (Supp Fig. S12). In brief, cell media was aspirated, and cells washed twice with serum-free RPMI and then PBS. One ml biotin solution [12 mg EZ-Link Sulfo-NHS-SS-Biotin (ThermoFisher) dissolved in 48 ml PBS] was gently added to cells and incubated with agitation at 4ºC for 25 minutes to biotinylate PM proteins. Cells were washed twice with quenching buffer (100 mM glycine in PBS) at 4ºC for 20 minutes, followed by a further two PBS washes. For negative controls (NEG), the labelling biotin solution was omitted, and cells incubated with PBS alone.


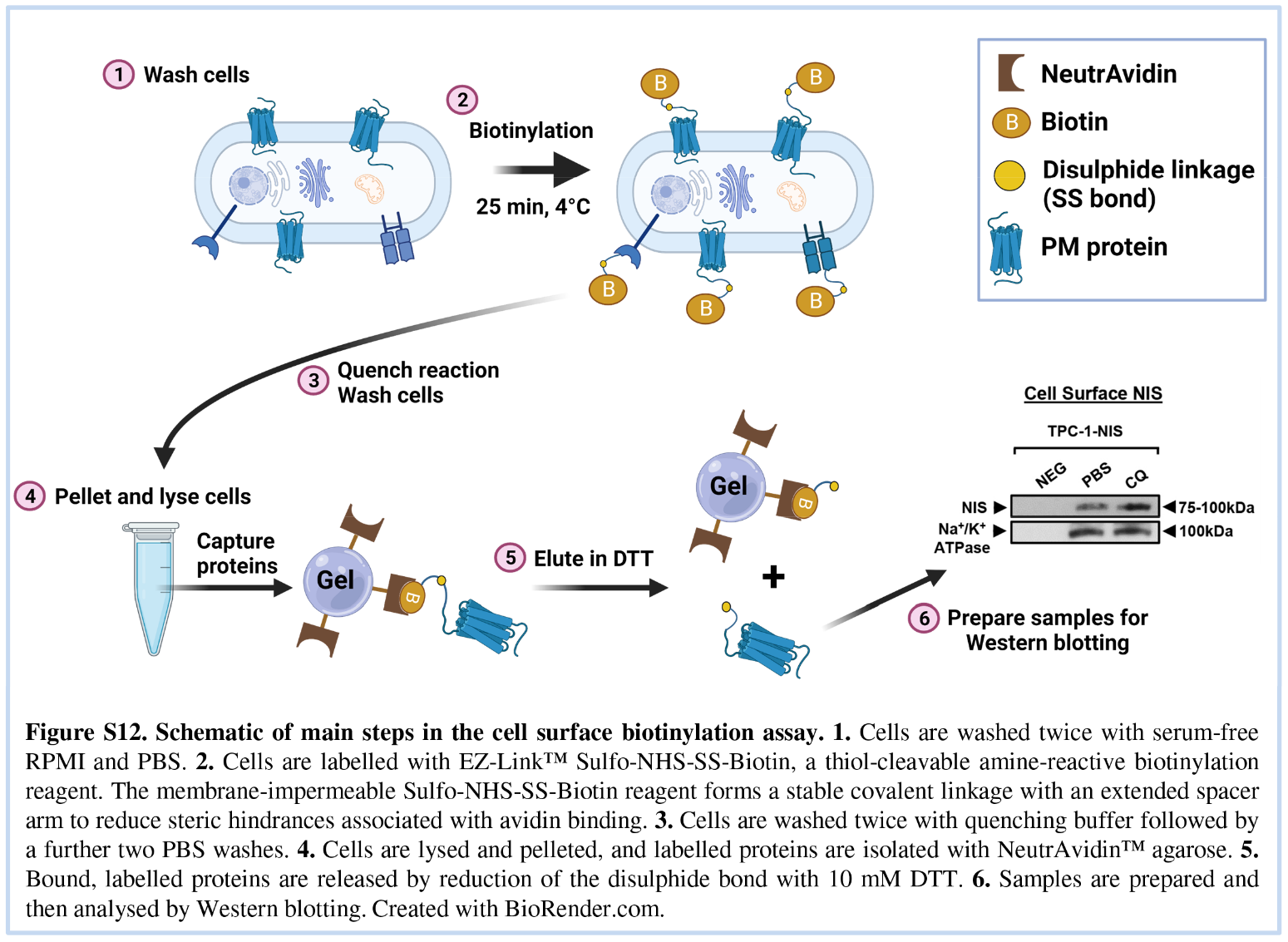
Cells were lysed [1% Triton-X in PBS containing protease and phosphatase inhibitor cocktails (Sigma-Aldrich)], centrifuged at 13,000 g for 15 minutes at 4ºC and supernatant protein concentrations quantified (Pierce™ BCA colorimetric assay, ThermoFisher). For total cell lysate controls, 40 μl of each supernatant was prepared with 5x protein loading buffer containing 12.5% β-mercaptoethanol (Sigma-Aldrich) and incubated at 37ºC for 30 minutes prior to storage at -20ºC. From the remaining cell lysates, equivalent volumes of protein for each experimental condition were used to isolate biotinylated proteins.

Immobilized NeutrAvidin gel (ThermoFisher) was incubated with each cell lysate overnight at 4ºC with end-over-end rotation. Samples were centrifuged at 1,000 g for 1 minute and supernatant discarded. After three washes with Triton-X lysis buffer to remove unbound protein, 100 μl SDS-PAGE sample buffer [62.5 mM Tris-HCl (pH 6.8), 3% SDS and 10% glycerol] containing 70 mM dithiothreitol (DTT) was incubated with the NeutrAvidin gel for 1 hour at room temperature with end-over-end rotation to elute PM proteins. Samples were centrifuged at 1,000 g for 1 minute and PM protein eluates collected and stored at -20ºC.

**TCGA and GEO datasets**

RNA-seq data for 59 normal thyroid and 501 PTC TCGA samples were analyzed (Broad GDAC Firehose, doi:10.7908/C11G0KM9). Normalised gene expression values were transformed as X=log2(X+1) where X represents the normalized fragments per kilobase transcript per million mapped reads (FPKM) values. Differential gene expression analysis was also performed using the GEO2R interactive web tool in GEO (9) to investigate endocytic genes in thyroid cancer, including analysis of the GEO dataset GSE60542 (8). Heatmaps were constructed using Morpheus (Broad Institute; https://software.broadinstitute.org/morpheus). Functional gene classification of TCGA RNA-seq data was performed using DAVID (10, 11) and ToppGene (12).

**Patient survival characteristics**

Receiver operating characteristic (ROC) curves were plotted in IBM SPSS Statistics (Version 29) and expression cut-off values calculated based on clinical sensitivity and specificity values nearest to 100%. Patients were then stratified into high and low expression groups for each individual gene, and survival characteristics determined [i.e. Kaplan-Meier (log-rank test), univariate and multivariate analyses). Using a continuous variable multivariate analysis, the Cox regression co-efficient was calculated for each gene to construct a 10, 20, 30 or 40 endocytic gene risk score classifier (Supplementary Table S3). A combined risk score for each patient was calculated according to the equation: risk score = ∑coefficient value ∗ expression (FPKM). Subsequent ROC curve analysis was performed for each multigene risk score classifier and patients grouped into high and low risk of recurrence using the calculated risk score cut-off point. Patient survival characteristics using the risk score were determined as described above.

**Statistical analyses**

All results were obtained from triplicate biological experiments unless otherwise indicated. For comparison between two groups, data were subjected to the Student’s t-test, and for multiple comparisons one-way ANOVA was used with either Dunnett’s or Tukey’s post-hoc test. Kruskal-Wallis and Spearman’s correlation tests were performed on non-parametric data. P-values were adjusted using the Benjamini-Hochberg FDR correction procedure to correct for multiple comparisons. Dunn’s multiple comparison post-hoc testing was used after Kruskal-Wallis tests to determine significance between datasets in groups of 3 or more. Fisher's exact test was used to determine the significance of nonrandom associations between two categorical variables. *P* < 0.05 was considered significant. All *P*-values reported from statistical tests were two-sided.

**Supplementary References**

1. Smith VE, Read ML, Turnell AS, Watkins RJ, Watkinson JC, Lewy GD*, et al.* A novel mechanism of sodium iodide symporter repression in differentiated thyroid cancer. Journal of Cell Science. **2009**;122(Pt 18):3393-402.

2. Read ML, Brookes K, Thornton CEM, Fletcher A, Nieto HR, Alshahrani M*, et al.* Targeting non-canonical pathways as a strategy to modulate the sodium iodide symporter. Cell Chem Biol. **2022**;29(3):502-16 e7.

3. Tiulpakov A, White CW, Abhayawardana RS, See HB, Chan AS, Seeber RM*, et al.* Mutations of Vasopressin Receptor 2 Including Novel L312S Have Differential Effects on Trafficking. Mol Endocrinol. **2016**;30(8):889-904.

4. Lan TH, Liu Q, Li C, Wu G, Lambert NA. Sensitive and high resolution localization and tracking of membrane proteins in live cells with BRET. Traffic. **2012**;13(11):1450-6.

5. Cerami E, Gao J, Dogrusoz U, Gross BE, Sumer SO, Aksoy BA*, et al.* The cBio cancer genomics portal: an open platform for exploring multidimensional cancer genomics data. Cancer Discov. **2012**;2(5):401-4.

6. Gao J, Aksoy BA, Dogrusoz U, Dresdner G, Gross B, Sumer SO*, et al.* Integrative analysis of complex cancer genomics and clinical profiles using the cBioPortal. Sci Signal. **2013**;6(269):pl1.

7. Grossman RL, Heath AP, Ferretti V, Varmus HE, Lowy DR, Kibbe WA*, et al.* Toward a Shared Vision for Cancer Genomic Data. N Engl J Med. **2016**;375(12):1109-12.

8. Tarabichi M, Saiselet M, Tresallet C, Hoang C, Larsimont D, Andry G*, et al.* Revisiting the transcriptional analysis of primary tumours and associated nodal metastases with enhanced biological and statistical controls: application to thyroid cancer. Br J Cancer. **2015**;112(10):1665-74.

9. Barrett T, Wilhite SE, Ledoux P, Evangelista C, Kim IF, Tomashevsky M*, et al.* NCBI GEO: archive for functional genomics data sets--update. Nucleic Acids Res. **2013**;41(Database issue):D991-5.

10. Huang da W, Sherman BT, Lempicki RA. Systematic and integrative analysis of large gene lists using DAVID bioinformatics resources. Nat Protoc. **2009**;4(1):44-57.

11. Huang da W, Sherman BT, Lempicki RA. Bioinformatics enrichment tools: paths toward the comprehensive functional analysis of large gene lists. Nucleic Acids Res. **2009**;37(1):1-13.

12. Chen J, Bardes EE, Aronow BJ, Jegga AG. ToppGene Suite for gene list enrichment analysis and candidate gene prioritization. Nucleic Acids Res. **2009**;37(Web Server issue):W305-11.
