# Supplementary material for: Combined Vorinostat and Chloroquine Inhibit Sodium Iodide Symporter Endocytosis and Enhance Radionuclide Uptake In Vivo
Source: Clin Cancer Res. Author manuscript; Available in PMC 2024 Apr 1. (PMC7615786; doi:10.1158/1078-0432.CCR-23-2043)
Supplement: Supplementary Table S2 [file EMS190879-supplement-Supplementary_Table_S2.pdf]

SUPPLEMENTARY TABLE S2

Clinical Variable	BRAF MUT, RAI-treated (*n* = 112)					BRAF-like, RAI-treated (*n* = 124)				
	n	Univariate *P*-value, HR (95% CI),		Multivariate *P*-value, HR (95% CI)		*n*	Univariate *P*-value, HR (95% CI)		Multivariate *P*-value, HR (95% CI)	
**Age, years**										
< 50<br>> 50	69<br>43	0.106	2.103 (0.854-5.181)	0.372	2.145 (0.401-11.46)	79<br>45	0.072	2.288 (0.929-5.634)	0.371	2.304 (0.370-14.347)
**Gender**										
Male<br>Female	40<br>72	0.741	0.849 (0.322-2.237)	0.645	0.784 (0.280-2.201)	39<br>90	0.994	0.996 (0.378-2.623)	0.807	0.878 (0.308-2.498)
**Stage**										
I + II<br>III + IV	64<br>48	0.099	2.157 (0.867-5.367)	0.932	1.078 (0.191-6.095)	70<br>54	0.114	2.085 (0.838-5.185)	0.968	1.038 (0.167-6.438)
**T stage**										
T1 + T2<br>T3 + T4	56<br>56	0.197	1.848 (0.727-4.694)	0.218	2.001 (0.663-6.037)	62<br>62	0.222	1.789 (0.704-4.544)	0.366	1.621 (0.569-4.622)
**Node stage**										
N0<br>N1	37<br>75	0.228	1.97 (0.654-5.939)	0.177	2.258 (0.693-7.361)	34<br>90	0.449	1.531 (0.508-4.617)	0.371	1.730 (0.521-5.748)
AP2A1										
Low<br>High	27<br>85	0.392	1.714 (0.499-5.882)	0.863	0.884 (0.216-3.616)	41<br>83	0.110	2.733 (0.797-9.390)	0.479	1.665 (0.406-6.819)
AP2A2										
Low<br>High	47<br>65	**0.022**	**4.220 (1.229-14.493)**	**0.009**	**6.310 (1.695-24.962)**	52<br>72	**0.025**	**4.097 (1.193-14.066)**	**0.018**	**4.806 (1.303-17.731)**
AP2B1										
Low<br>High	57<br>55	0.230	1.771 (0.697-4.498)	0.51	0.677 (0.212-2.163)	65<br>59	0.167	1.929 (0.759-4.902)	0.834	0.889 (0.297-2.667)
AP2M1										
Low<br>High	66<br>46	0.332	0.619 (0.235-1.630)	0.66	0.760 (0.224-2.578)	75<br>49	0.411	0.666 (0.253-1.753)	0.559	0.703 (0.215-2.296)
AP2S1										
Low<br>High	46<br>66	0.087	0.452 (0.182-1.123)	0.051	0.295 (0.086-1.006)	51<br>73	0.082	0.445 (0.179-1.107)	**0.044**	**0.295 (0.090-0.969)**

Multivariate analysis of AP2 genes in THCA. *n*, number; HR. hazard ratio; CI, confidence interval. *P*-values in bold were less than 0.05. Some patients in the BRAF MUT, RAI treated (*n* = 13) and BRAF-like, RAI-treated (*n* = 13) cohorts were not included in univariate and multivariate analysis due to missing clinical variables.
